# Characterization of trace elements in thermal and mineral waters of Greece

**DOI:** 10.1007/s11356-023-27829-x

**Published:** 2023-06-03

**Authors:** Lorenza Li Vigni, Kyriaki Daskalopoulou, Sergio Calabrese, Konstantinos Kyriakopoulos, Sergio Bellomo, Lorenzo Brusca, Filippo Brugnone, Walter D’Alessandro

**Affiliations:** 1 DiSTeM, University of Palermo, Via Archirafi 36, Palermo, Italy; 2 Institute of Geosciences, University of Potsdam, Karl-Liebknecht-Str. 24-25, Potsdam-Golm, Germany; 3 Physics of Earthquakes and Volcanoes, GeoForschungs Zentrum, Helmholtzstraße 6/7, Potsdam, Germany; 4 Istituto Nazionale di Geofisica e Vulcanologia, Sezione di Palermo, Via Ugo La Malfa 153, Palermo, Italy; 5 Faculty of Geology and Geoenvironment, National and Kapodistrian University of Athens, Ano Ilissia, Panestimioupolis, Greece

**Keywords:** Hydrogeochemistry, Trace elements, Natural contaminants, Water-rock interaction, Thermal waters, Mineral waters, Greece

## Abstract

Natural thermal and mineral waters are widely distributed along the Hellenic region and are related to the geodynamic regime of the country. The diverse lithological and tectonic settings they are found in reflect the great variability in their chemical and isotopic composition. The current study presents 276 (published and unpublished) trace element water data and discusses the sources and processes affecting the water by taking into consideration the framework of their geographic distribution. The dataset is divided in groups using temperature- and pH-related criteria. Results yield a wide range of concentrations, often related to the solubility properties of the individual elements and the factors impacting them (i.e. temperature, acidity, redox conditions and salinity). Many elements (e.g. alkalis, Ti, Sr, As and Tl) present a good correlation with temperature, which is in cases impacted by water rock interactions, while others (e.g. Be, Al, Cu, Se, Cd) exhibit either no relation or an inverse correlation with T possibly because they become oversaturated at higher temperatures in solid phases. A moderately constant inverse correlation is noticed for the vast majority of trace elements and pH, whereas no relationship between trace element concentrations and Eh was found. Seawater contamination and water-rock interaction seem to be the main natural processes that influence both salinity and elemental content. All in all, Greek thermomineral waters exceed occasionally the accepted limits representing in such cases serious harm to the environment and probably indirectly (through the water cycle) to human health.

**Supplementary Information:**

The online version contains supplementary material available at 10.1007/s11356-023-27829-x.

## Introduction

The term “trace elements” refers to the relative abundance of some elements in different media. Specifically, the term indicates chemical elements that in natural systems are characterized by concentrations generally lower than 1 mg L^−1^ in solution or 0.1% in solids (Gaillardet et al. 2003; Koller and Saleh 2018). Indeed, some elements that are considered “trace” in one media are “major” in another; e.g. Al is a major constituent in rocks but is found at trace levels in waters (Gaillardet et al. 2003). Although present in lower amounts, they play significant roles in natural systems (Kabata-Pendias 2010), such as geological processes (e.g. Geibert 2018; Yan et al. 2021) and biochemical reactions (e.g. Gomes et al. 2014).

Some trace elements are essential for organisms (e.g. Mn, Fe, Co, Ni, Cu, Zn, Mo, Se, I for the human body), but excess in their bioavailable concentrations can be toxic (Wada 2004). In other cases, their depleted levels may lead to adverse health effects on living organisms. For instance, deficiency in I induces thyroid disorders in humans (e.g. goitre, carcinoma, etc. — Zimmermann and Boelaert 2015), and deficiency in Se can be responsible for various disorders like Keshan and Kashin-Beck diseases (Fordyce 2013). Similar to humans, also for animals and plants, deficiency and, more frequently, excess of potentially toxic trace elements can be detrimental.

Harmful levels of trace elements have often been related to anthropogenic processes (pollution), and plenty of examples can be found in literature (e.g. Abou Seedo et al. 2017; Jahan and Strezov 2019). One of the most complete studies was the *Kola Ecogeochemistry Project*, which investigated multiple media (mosses, humus, topsoil, mineral soil, groundwater, surface water, rainwater, snow, etc.) on a large area (188,000 km^2^) around several huge ore extractions and processing locations (de Caritat and Kofoed 1995). Results revealed a wide range of concentrations, often spanning over several orders of magnitude for the majority of the analysed elements. Very low values were recognised in pristine areas, while abnormally high values were identified in the most polluted zones evidencing the strong impact of human activities (Reimann et al. 1998; Reimann and de Caritat 2005).

Even though abnormal trace element levels often reflect anthropogenic factors, harmful trace element levels may also derive from natural processes (contamination) (e.g. Brugnone et al. 2020; Calabrese et al. 2016; D’Alessandro et al. 2017; 2020; Gagliano et al. 2019; Rognerud and Fjeld 2001). For example, volcanic activity is a strong contributor of trace elements to the atmosphere (Calabrese et al. 2011; Liotta et al. 2021) and the ocean (Elderfield and Shultz 1996).

Water is fundamental for life, and the greatest management problems derive from the quantity and quality of this resource. Quality problems in surface- and ground- water may originate both from major components and trace elements. The latter include heavy metals and non-metals, whose abundance is related to the rock type the waters interact with (Banks et al. 1995; Li Vigni et al. 2022a) and to pollution deriving from human activity (Gaillardet et al. 2003).

The main natural process that favours the solubility of trace elements is water–rock interaction. Higher temperatures and low pH values generally favour this process, and hydrothermal activity is mostly related to both hot and acidic fluids. Therefore, geothermal energy, although included among the “green” energies, has long been considered a not always clean form of energy (Ellis 1978). Surface- and ground- water may be contaminated by hydrothermal fluids exceeding safe limits of F, B and As and many heavy metals (Ellis 1978; Aiuppa et al. 2006; Baba and Armannsson 2006; Guo et al. 2008; 2009; Bagnato et al. 2009; Wang et al. 2018).

Acidic fluids are not necessarily correlated with thermal anomalies, and acid waters may be related to the ascent of endogenous gases like CO_2_ and H_2_S (e.g. Aiuppa et al. 2021; Daskalopoulou et al. 2022; Li Vigni et al. 2022a). Waters, where H_2_S is oxidized producing H_2_SO_4_, may attain very low pH values (< 5), therefore strongly enhancing water–rock interaction processes and reaching high concentrations in trace elements (Nordstrom et al. 2009). Similarly, also oxidation of sulfide minerals may produce highly acidic and metal-laden waters (Acid Mine Drainage; Blowes et al. 2003). These conditions are rarely achieved but, on the contrary, high concentrations of dissolved CO_2_ are common. Strong geogenic degassing is mostly connected to volcanically and tectonically active areas (Tamburello et al. 2018), which are frequently accompanied by widespread CO_2_-rich groundwater with a bubbling-free gas phase (Chiodini et al. 2000; Randazzo et al. 2021). These waters may sometimes reach very high contents in trace elements (Aiuppa et al. 2003; D’Alessandro et al. 2011). Note that areas of accumulation and surface release of CO_2_-charged waters have been used as natural equivalents to study the impact of leaking carbon storage systems on hydrosphere (Pearce 2004).

Guideline values of trace elements in natural waters, destined for drinking use, have been established to safeguard human health from environmental pollution and natural contamination. The drinking water quality limits are regulated by the European Union with the Directive 20/2184/EC (Council Directive 2020). It has to be underscored that the quality of mineral water is regulated by a different Directive (2003/40/EC), which has different limits for some elements (e.g. F, Mn.). Greek national law regulating water quality follows the EU directives. In general, not all trace elements are regulated by law, and, often, guideline values from the World Health Organization (WHO) and the US Environmental Protection Agency (USEPA) are missing even for elements that are considered toxic at high concentrations like Bi, Tl or V.

Greece, being a geodynamically active region, is widely affected by hydrothermalism (Lambrakis and Kallergis 2005), and numerous gas-charged mineral springs are found all along the Hellenic territory (Daskalopoulou et al. 2018; 2019).

The widespread thermomineral water resources have long been the targets of scientific studies although almost always limited to major constituents and few minor elements (Li, B, Br, I). Some of these studies, starting with the book of Landerer (1843), covered the entire Greek territory (Pertessis 1937; Lambrakis and Kallergis 2005; Athanasoulis et al. 2009; Lambrakis et al. 2013a). However, apart from those of our research group that comprise data included in the present paper (D’Alessandro et al. 2011, 2014, 2017, 2018; Li Vigni et al. 2021), only a limited number of papers reported analyses of trace elements from these waters (e.g. Lambrakis and Stamatis 2008; Stamatakis et al. 2009; Kelepertsis et al. 2009; Tziritis and Kelepertzis 2011; Lambrakis et al. 2013b; Katsanou et al. 2012, 2022), and sometimes, they are limited to single elements (e.g. B — Dotsika et al. 2006; Cr — Kaprara et al. 2015; As — Kouras et al. 2007).

**Fig. 1:**
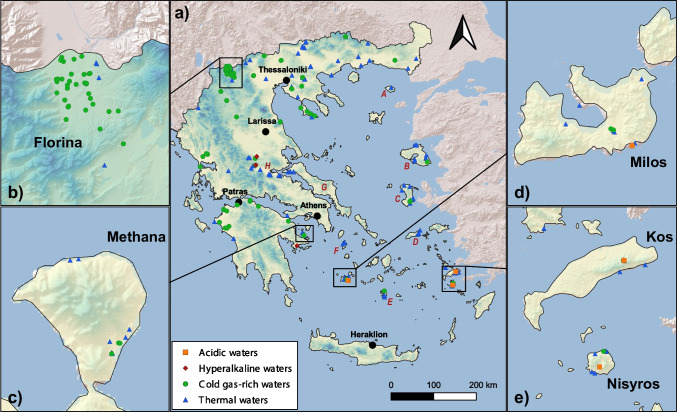
Geographic distribution of the sampling sites. Insets are enlarged areas with high density of sampling sites. (**a**) Samothraki; (**b**) Lesbos; (**c**) Chios; (**d**) Ikaria; (**e**) Santorini; (**f**) Kythnos; (**g**) Euboia; (**h**) Othrys and Sperchios Basin (modified from Li Vigni et al. 2022a, published under Creative Commons licence. To view a copy of this licence, visit http://creativecommons.org/licenses/by/4.0/). Basemap by *ESRI* maps

In the period from October 2004 to March 2020, nearly 300 water samples were collected along the Greek territory (Fig. 1) and analysed for their major, minor and trace constituents, and the isotopic composition of water. Results of the major constituents’ chemistry and water isotope composition are discussed in a companion paper (Li Vigni et al. 2022a). This paper focuses on the analytical results of a large suite of trace elements and includes previously published data to complete the coverage of the whole Hellenic territory. Although samples have been collected over a long period, the sampling methods remained constant, and the analyses were always made in the same laboratory, ensuring comparable results. The present paper summarizes the geographical distribution of trace elements in the thermal and mineral waters of Greece and analyses the possible sources and geochemical processes that control their concentrations in the studied waters. The possible impact on human health of some elements is also briefly discussed.

## Study area

The northward subduction of the African plate under the Eurasian at a rate of > 20 mm a^−1^ (McClusky et al. 2000) is the dominant tectonic process taking place along the Hellenic region. This movement resulted in the formation of a back arc basin, the Aegean Sea and the generation of the South Aegean Active Volcanic Arc (SAAVA-Fytikas et al. 1984). Moreover, tectonic structures of N-S crustal extension (Mercier 1981) and the westward motion of the Anatolian plate along the strike-slip North Anatolian Fault — NAF (Pavlides and Caputo 1994) — contributed to Greece’s complex, though interesting, geodynamic regime. This tectonic situation, along with the associated crustal deformation of the region (Jolivet et al. 2010), makes Greece one of the most seismically active and rapidly deforming areas worldwide (e.g. Le Pichon et al. 2001; Taymaz et al. 1991; Pavlides et al. 2010).

Based on the aforementioned tectonic setting, Mountrakis (1985, 2010) identified three isopic zones, which are (from east to the west): (i) Hellenic Hinterland, which includes Rhodope and Serbo-Macedonian Massifs representing a Precambrian-Silurian continental crust affected by metamorphism; (ii) Internal Hellenides, which consists of six zones (Circum-Rhodope zone, Vardar zone, Pelagonian zone, Subpelagonian zone, Pindos zone and Attico-Cycladic zone) characterized by obducted ophiolites and mostly deep-sea sediments; (iii) External Hellenides that consist of the Parnassos zone, Gavrivo-Tripoli zone, Ionian zone and Paxos zone, corresponding to a neritic continental depositional environment.

This regime, along with the high terrestrial heat flow (Fytikas and Kolios 1979), favoured the existence of numerous thermal and cold manifestations (Daskalopoulou et al. 2019; Li Vigni et al. 2022a). Moreover, the geo-tectonic setting and the presence of SAAVA also had an impact on the chemical characteristics of the waters. For instance, geothermal activity in Sousaki (central Greece) has resulted in alteration outcrops rich in highly soluble sulfate minerals with enhanced trace metal content (D’Alessandro et al. 2020), while the volcanism and the fault system of Milos Island are responsible for the metalliferous mineralization (Fytikas 1977; Kilias et al. 2001). Further mineral deposits in Greece are connected to recent (Miocene–Pliocene) volcanism (Melfos and Voudouris 2012; Pe-Piper et al. 2014; Skarpelis 2002). Examples can be found in Chalkidiki (Northern Greece), where the related high heat flow values and tectonics favoured the development of Au–Ag–Pb–Zn–Cu sulfide deposits with pyrite, arsenopyrite, sphalerite and galena as their main ore minerals (Hahn et al. 2012). Furthermore, the two extensive ophiolitic belts belonging to the Internal Hellenides are affected by peculiar mineralisations. These build up sometimes exploitable chromite and Fe–Cu–Co–Zn-type sulfide ore deposits (Tzamos et al. 2016; Eliopoulos et al. 2020).

## Materials and methods

In the period 2004–2020, 276 natural water samples were collected along the Hellenic territory (Fig. 1). The analyses of 93 of these samples were previously published (D’Alessandro et al. 2011, 2014, 2017, 2018; Li Vigni et al. 2021), while the remaining samples are unpublished. For all samples, physicochemical parameters (temperature, pH, Eh and electric conductivity) were measured in situ, with Eh being measured only in about two-thirds of the samples. Total alkalinity was determined in situ by titration with 0.1N HCl on unfiltered samples. Water samples were filtered (0.45 μm MF-Millipore cellulose acetate filters), and for the determination of metals, they were acidified with ultrapure concentrated HNO_3_ and stored in HDPE bottles. All analyses were performed at the laboratories of the *Istituto Nazionale di Geofisica e Vulcanologia* (INGV) of Palermo.

Major ions (Na^+^, K^+^, Mg^2+^, Ca^2+^, F^−^, Cl^−^, NO_3_^−^ and SO_4_^2−^) were determined by ion chromatography. Further analytical and methodological information can be found in Li Vigni et al. (2022a).

Trace elements (Al, As, B, Ba, Be, Ba, Cd, Co, Cr, Cs, Cu, Fe, Li, Mn, Mo, Ni, Pb, Rb, Sb, Se, Sr, Ti, Tl, U, V and Zn) were analysed by ICP-MS (Agilent 7500ce) equipped with Micromist nebuliser, Scott double-pass spray chamber, one-piece quartz torch and Octopole Reaction System (ORS) to reduce molecular interferences on the masses of investigated analytes. External calibrations were performed with standard solutions obtained by mixing and diluting multi- and single-element work solutions (100 mg L^−1^ and 1000 mg L^−1^, CertiPUR ICP Standards Merck). The calibration routine was done on selected isotopes for each element with 11 calibration points (Supplementary materials) prepared daily in 10-mL polyethylene tubes by dilution with 2% nitric acid solution, treated as a blank solution. Element contents in the analysed samples were calculated using the spectrometer software (ICP Mass Hunter, version B.01.01). The sensitivity variations were monitored by ^103^Rh, ^115^In and ^185^Re with 10 μg L^−1^ concentration as an internal standard added directly online. The precision of analysis, checked by running 5 replicates, was always within ± 15%. Data accuracy was evaluated by analysing standard reference materials (Spectrapure Standards SW1 and 2, NIST 1643e, TM 61.2 Environment Canada, and SLRS4 NRC Canada) and was always within 10%. All solutions were prepared daily with deionised water to 18.2 MΩ produced with Elix System, Integral 5 by Milli-Q, Millipore. The acids used are ultra-pure grade by JT Baker.

Silica (SiO_2_) and high concentrations of Li, B, Sr, Fe and Mn were determined with ICP-OES (Jobin Yvon Ultima 2).

Total dissolved solids (TDS) are here intended as the sum of all determined major ions plus silica.

Factor analysis (FA) is a multivariate method used to describe the relationships among a set of observable variables with a smaller number of unobservable variables called factors. It works very well when the studied variables are highly correlated and the goal is to group them according to the extracted factors. For instance, if all the variables in one group are highly correlated among them and have little correlation with the variables in the remaining groups, each group can represent a factor. Therefore, FA is used to reveal relationships between the original variable and unobserved underlying dimensions (Wang 2009).

In the FA analysis of the Greek thermal and mineral waters, also temperature, electric conductivity, pH, major ions and silica from Li Vigni et al. (2022a) have been considered as input variables.

Results were obtained in three steps (SPSS version 21 for windows): (i) calculation of the covariance/correlation matrix in order to determine whether the dataset is suitable for FA; (ii) identification of the number of factors to be considered on the basis of the scree plot; (iii) rotation of the factors with the aim to increase their interpretability by maximising loadings on individual factors (varimax rotation).

## Results

**Fig. 2:**
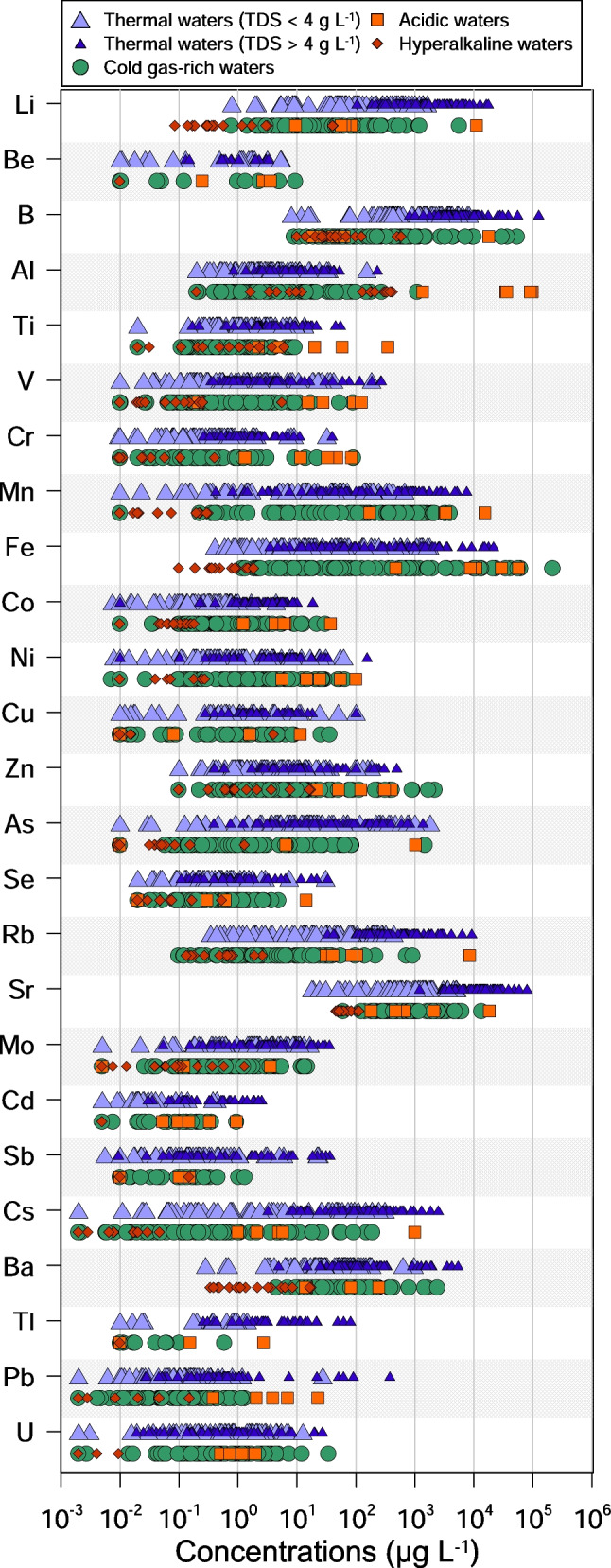
Trace element concentrations in the thermal and mineral waters of Greece

**Fig. 3:**
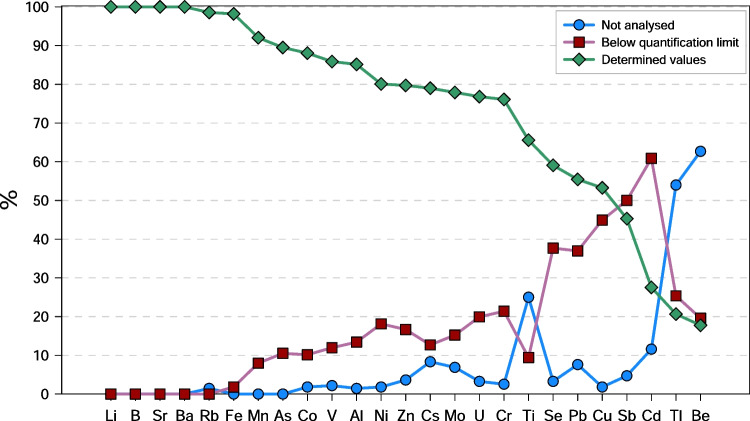
Percentage of samples with concentrations below quantification limit (red squares) or not analysed (blue circles) for some specific element. Green diamonds indicate the percentage of samples having the concentration of the element determined

Collected waters show a wide range of values both for their physical–chemical parameters and their chemical composition (Table 1). Temperatures were measured at the spring outlet and range from 6.5 to 98 °C. pH values vary from 1.96 to 11.98, and Eh ranges from −391 to 331 mV. TDS concentrations span between 0.06 and 43 g L^−1^. The wide ranges of the physicochemical parameters are reflected in the great variety of chemical compositions of the waters, previously described by Li Vigni et al. (2022a). As a consequence, also the concentrations of trace elements span many orders of magnitude (Fig. 2) from three up to more than seven (e.g. Fe). Roughly, they can be subdivided into elements never exceeding 10 µg L^−1^ (Be and Cd), 100 µg L^−1^ (Cr, Co, Cu, Mo, Sb, Se, Tl and U), 1000 µg L^−1^ (Ni, Ti, Pb and V), 10,000 µg L^−1^ (As, Ba, Cs, Rb and Zn), 100,000 µg L^−1^ (Br, Li, Mn and Sr) and more than 100,000 µg L^−1^ (Al, B and Fe). Among the considered elements, only seven (Li, B, Mn, Fe, As, Sr and Ba) were analysed in all samples, while less than 10% of the samples were not analysed for one of the following 14 elements: Al, Cs, Co, Cr, Cu, Mo, Ni, Pb, Rb, Sb, Se, U, V and Zn (Fig. 3). Few elements, i.e. Cd, Ti, Tl, and Be, were analysed less frequently (11.6%, 25.0%, 54.0% and 62.7% of not analysed samples, respectively). Furthermore, sometimes in the analysed samples, concentrations were below quantification limit as also shown in Table 1. Taking into consideration both samples with one not analysed element and samples with concentrations of the same element below detection limit, only four elements (Li, B, Sr and Ba) show no missing values in the whole dataset, and only four (Sb, Cd, Tl and Be) include less than 50% of determined values (Fig. 3).

**Table 1:** Statistical parameters of trace elements in Greek thermal and mineral waters

Elements	L.O.Q	Thermal waters (TDS < 4 g L^−1^)					Thermal waters (TDS > 4 g L^−1^)				
µg L^−1^		Min	25%	Median	75%	Max	Min	25%	Median	75%	Max
Li	0.1	0.791	37.4	76.2	336	1625	104	511	1333	3140	17,630
Be	0.01	*b.q.l*	*b.q.l*	0.056	1.31	5.49	*b.q.l*	*b.q.l*	0.13	0.779	3.32
B	2	8.12	401	937	2284	8304	808	3674	5127	8465	126,402
Al	0.2	*b.q.l*	1.13	2.47	4.72	153	*b.q.l*	0.200	5.31	11.7	230
Ti	0.02	*b.q.l*	0.462	1.12	3.16	13.5	*b.q.l*	*b.q.l*	2.19	4.92	54.3
V	0.01	*b.q.l*	0.095	0.311	2.81	184	*b.q.l*	0.675	2.01	4.76	269
Cr	0.01	*b.q.l*	*b.q.l*	0.165	0.509	32	*b.q.l*	0.410	1.06	2.10	39.4
Mn	0.01	*b.q.l*	0.371	7.21	54.2	669	*b.q.l*	24.9	99.8	723	7569
Fe	0.01	0.406	4.12	14.3	76.0	1774	*b.q.l*	32.3	178	987	21,593
Co	0.01	0.007	0.014	0.174	0.456	4.44	*b.q.l*	1.25	2.15	4.58	18.7
Ni	0.01	0.008	0.103	0.472	1.97	61.8	*b.q.l*	0.146	2.82	7.06	154
Cu	0.01	*b.q.l*	*b.q.l*	0.011	0.735	100	*b.q.l*	*b.q.l*	0.339	2.52	99.7
Zn	0.1	*b.q.l*	0.827	2.71	10.9	183	*b.q.l*	0.100	4.06	12.0	492
As	0.01	*b.q.l*	2.38	9.47	27.0	1819	*b.q.l*	8.86	34.3	190	1369
Se	0.02	*b.q.l*	*b.q.l*	0.211	0.504	30.6	*b.q.l*	*b.q.l*	0.220	1.06	33.1
Rb	0.1	0.345	3.81	18.8	85.2	438	33	169	342	882	9227
Sr	0.1	18.0	338	619	1341	5108	1199	6362	11,340	17,239	79,998
Mo	0.005	*b.q.l*	0.406	1.82	4.20	16.6	*b.q.l*	0.345	2.32	5.35	35.3
Cd	0.005	*b.q.l*	*b.q.l*	0.005	0.016	0.44	*b.q.l*	*b.q.l*	0.005	0.124	2.53
Sb	0.01	0.006	0.010	0.069	0.326	23.6	0.009	0.010	0.117	0.942	35.9
Cs	0.002	*b.q.l*	0.160	1.71	38.5	310	3.24	53.6	175	293	2493
Ba	0.02	0.283	17.0	30.2	70.6	961	4.91	64.5	98.3	225	5399
Tl	0.01	*b.q.l*	*b.q.l*	0.01	0.327	1.44	*b.q.l*	*b.q.l*	1.99	6.97	80.9
Pb	0.002	*b.q.l*	*b.q.l*	0.023	0.139	27.4	*b.q.l*	*b.q.l*	0.142	0.632	375
U	0.002	*b.q.l*	0.075	0.62	2.04	12.7	*b.q.l*	0.044	0.253	0.982	26.9

Elements	Cold gas-rich waters					Acid waters					Hyperalkaline waters				
µg L^−1^	Min	25%	Median	75%	Max	Min	25%	Median	75%	Max	Min	25%	Median	75%	Max
Li	0.773	7.22	22.3	84.1	5683	9.65	56.9	66.2	85.2	11,278	0.086	0.297	0.380	1.61	41.4
Be	*b.q.l*	*b.q.l*	0.01	0.104	9.51	0.252	2.10	3.03	3.40	3.61	*b.q.l*	*b.q.l*	*b.q.l*	*b.q.l*	*b.q.l*
B	9.008	55.4	211	759	54,630	18.8	22.6	40.6	64.0	18,224	10.1	29.5	40.5	68.8	593
Al	b.q.l	0.640	1.77	8.69	1101	1380	35,480	36,882	92,673	100,246	*b.q.l*	8.23	180	337	421
Ti	*b.q.l*	0.300	0.581	1.60	9.31	2.27	5.09	20.6	59.1	354	*b.q.l*	*b.q.l*	0.234	1.08	6.18
V	*b.q.l*	0.155	0.360	1.40	89.3	0.215	15.8	28.1	93.0	126	*b.q.l*	0.021	0.043	0.145	5.63
Cr	*b.q.l*	0.039	0.191	0.424	90.7	1.34	11.8	33.6	48.3	85.3	0.009	0.010	0.01	0.025	0.406
Mn	*b.q.l*	7.97	77.3	519	3969	174	3227	3336	3423	15,649	*b.q.l*	*b.q.l*	0.018	0.167	0.316
Fe	1.171	9.74	55.9	1013	21,7637	480	8889	11,100	30,020	58,385	*b.q.l*	0.360	0.847	1.47	1.88
Co	*b.q.l*	0.189	0.487	1.56	30.3	1.25	4.34	6.17	6.49	37.7	*b.q.l*	0.064	0.098	0.129	0.183
Ni	0.007	0.466	1.90	7.00	64.8	5.69	14.9	24.9	55.1	101	*b.q.l*	*b.q.l*	*b.q.l*	0.058	0.277
Cu	*b.q.l*	*b.q.l*	0.245	1.42	35.9	b.q.l	*b.q.l*	0.082	1.61	11.7	*b.q.l*	*b.q.l*	*b.q.l*	*b.q.l*	4.06
Zn	*b.q.l*	1.35	5.97	22.0	2189	22.4	51.1	123	309	406	*b.q.l*	0.156	0.839	2.15	17.4
As	*b.q.l*	0.218	1.02	2.82	1489	*b.q.l*	*b.q.l*	*b.q.l*	6.63	1042	0.009	0.010	0.036	0.051	1.31
Se	*b.q.l*	*b.q.l*	0.167	0.793	4.93	*b.q.l*	*b.q.l*	0.304	0.619	14.7	*b.q.l*	*b.q.l*	*b.q.l*	0.043	0.534
Rb	0.099	1.00	4.81	19.9	922	31.9	40.9	86.4	106	8618	0.134	0.331	0.645	0.753	2.64
Sr	60.51	340	566	1142	13,530	183	475	684	2126	18,727	46.2	52.6	59.3	81.8	114
Mo	*b.q.l*	0.089	0.344	1.08	15.2	*b.q.l*	*b.q.l*	0.113	0.123	3.61	*b.q.l*	0.009	0.059	0.112	1.30
Cd	*b.q.l*	*b.q.l*	0.005	0.025	0.953	0.054	0.098	0.149	0.335	0.958	*b.q.l*	*b.q.l*	*b.q.l*	*b.q.l*	*b.q.l*
Sb	*b.q.l*	*b.q.l*	0.01	0.025	1.32	*b.q.l*	*b.q.l*	*b.q.l*	0.102	0.152	*b.q.l*	*b.q.l*	*b.q.l*	*b.q.l*	0.150
Cs	*b.q.l*	*b.q.l*	0.11	0.739	191	1.01	2.13	4.96	5.86	1006	*b.q.l*	*b.q.l*	0.008	0.019	0.047
Ba	4.501	40.0	73.6	136	2421	14.2	14.7	15.0	83.2	247	0.342	0.550	1.25	5.09	17.7
Tl	*b.q.l*	*b.q.l*	0.01	0.017	0.591	*b.q.l*	*b.q.l*	0.083	0.806	2.75	*b.q.l*	*b.q.l*	*b.q.l*	*b.q.l*	*b.q.l*
Pb	*b.q.l*	*b.q.l*	0.026	0.173	1.30	0.386	2.09	3.96	7.06	23.0	*b.q.l*	*b.q.l*	*b.q.l*	0.003	0.151
U	*b.q.l*	0.098	0.379	1.35	34.4	0.513	0.737	1.19	1.40	2.00	*b.q.l*	*b.q.l*	*b.q.l*	*b.q.l*	0.010

Values are expressed as µg L^−1^

L.O.Q. limit of quantification, b.q.l. below quantification limit

### Factor analysis

Factor analysis was applied on a reduced dataset excluding hyperalkaline (pH > 11) and highly acidic (pH < 5) waters. Furthermore, 17 samples were left out because one or more variables were missing, and 6 variables (Eh, Be, Ti, Cd, Tl and Pb) were not considered since in many samples, they were not analysed. Most of the variables (including the electrical conductivity and the major ions) approached a log-normal distribution, and therefore, FA was applied to log-transformed variables. Only temperature and pH were not log-transformed. The final dataset consisted of 236 samples and 31 variables. Results are shown in Table. 2.

**Table 2:** Factor analysis

	Factors				
	1	2	3	4	5
T	0.55	−0.05	−0.21	**0.56**	0.04
pH	−0.13	** −0.81**	−0.28	0.02	0.13
El. Cond	**0.96**	0.15	0.14	0.11	0.01
Ca	**0.71**	0.48	0.31	−0.03	−0.20
Mg	**0.70**	0.43	0.39	−0.21	−0.20
Na	**0.96**	−0.03	0.04	0.21	0.10
K	**0.88**	0.20	0.18	0.27	0.05
Alk	0.02	**0.54**	−0.10	0.15	−0.27
Cl	**0.97**	0.002	0.12	0.10	0.09
SO_4_	**0.74**	−0.02	0.23	−0.005	−0.04
SiO_2_	−0.04	0.25	−0.05	**0.53**	0.29
Li	**0.71**	0.27	−0.06	0.57	−0.04
B	**0.69**	−0.03	−0.15	0.42	0.09
Al	0.02	0.05	0.09	−0.06	**0.36**
V	0.26	−0.07	**0.68**	0.16	0.02
Cr	0.34	0.01	**0.55**	0.06	0.08
Mn	0.17	**0.69**	0.06	0.10	0.35
Fe	0.11	**0.67**	−0.09	0.19	0.31
Co	0.38	**0.61**	0.48	−0.03	0.10
Ni	−0.08	0.38	**0.62**	−0.10	0.05
Cu	0.06	−0.003	**0.60**	−0.16	0.20
Zn	−0.19	0.16	**0.33**	0.01	0.17
As	0.44	0.21	0.18	**0.52**	−0.27
Se	0.06	−0.01	**0.38**	0.09	−0.08
Rb	**0.72**	0.22	0.04	0.56	−0.04
Sr	**0.85**	0.39	0.08	0.15	−0.04
Mo	0.19	−0.35	0.27	0.23	−0.12
Sb	0.23	−0.02	0.20	**0.52**	−0.18
Cs	**0.64**	0.16	−0.05	0.59	−0.22
Ba	0.32	**0.54**	0.07	0.19	0.03
U	−0.04	0.04	**0.55**	−0.08	−0.46
Explained variability %	28.5	12.0	9.6	8.8	3.5

Values in bold indicate the variables that characterise each factor

Five factors were extracted explaining 62.6% of the dataset variance. The first factor (28.5% variance) shows very high loadings (> 0.8) for electric conductivity, Na, K, Cl and Sr, while Ca, Mg, SO_4_, Li, B, Rb and Cs all exceeded 0.6. The highest loadings (0.61–0.69) on the second factor (12.0% of variance) are shown by Mn, Fe, and Co. Alkalinity and Ba show moderate loadings (> 0.5) on the second factor, while pH shows a high inverse one (−0.81). In the third factor (9.6% of variance), V, Cr, Ni, Cu and Ba show loadings between 0.55 and 0.68. Although not very high, Zn (0.38) and Se (0.33) present their highest loadings on this factor. The fourth factor (8.8% of variance) includes temperature, SiO_2_, As and Sb and exhibits moderate loadings (> 0.5). Finally, the fifth factor (3.5% of variance) does not show high loadings for any variable (−0.46 to 0.36), and only Al shows his highest loading on this factor (0.36).

## Discussion

To better discuss the major ion composition of the sampled thermal and mineral waters, Li Vigni et al. (2022a) subdivided the whole dataset into groups and subgroups. The first discrimination was made based on the measured pH values and resulted in the identification of two groups characterized by extreme values: hyperalkaline (pH > 11) and highly acidic (pH < 5). The remaining samples were subdivided into cold (< 23 °C) and thermal waters. Such subdivision was applied in an attempt to obtain moderately homogeneous groups referring sometimes to specific processes. For example, hyperalkaline waters of the presented dataset are always related to serpentinization processes (Li Vigni et al. 2021), while highly acidic waters are connected with hydrothermal sulfide oxidation (Li Vigni et al. 2022a). Thermal waters were further subdivided into saline (TDS > 4 g L^−1^) and non-saline waters.

While the composition of the hyperalkaline and strongly acidic waters can be attributed prevailingly to specific processes (serpentinization of ultramafic rocks for the former and oxidation of hydrothermal H_2_S for the latter), the composition of the remaining waters may be ascribed to many concurring factors. The results of FA indicate at least four important factors impacting the composition of the thermal and mineral waters (Table 2). The first factor is salinity, which depends mainly on two processes: seawater contamination and water–rock interaction (Li Vigni et al. 2022a). The influence of the dissolution of evaporitic rocks is trivial (Li Vigni et al. 2022a). The second is an acidity factor, where water pH is inversely related to many elements. This factor can be also attributed to the amount of CO_2_ dissolved in the water. Carbon dioxide after dissolution reacts with water following the reactions:

1 $${\mathrm{CO}}_{2}+{\mathrm{H}}_{2}\mathrm{O}\leftrightarrow {\mathrm{H}}_{2}{\mathrm{CO}}_{3}$$

2 $${\mathrm{H}}_{2}{\mathrm{CO}}_{3}\leftrightarrow {\mathrm{H}}^{+}+{\mathrm{HCO}}_{3}^{-}\leftrightarrow 2{\mathrm{H}}^{+}+{{\mathrm{CO}}_{3}}^{2-}$$

The inverse relationship between CO_2_ content and pH is apparent, justifying the strong negative loading of the latter associated with the positive loading of total alkalinity on the third factor.

The third factor groups many transitional elements mostly related to the weathering of the ophiolitic rocks that are widely outcropping in Greece. Finally, the fourth factor can be considered temperature-related as it mainly depends on hydrothermal activity.

### Relationship with the physical-chemical parameters

**Fig. 4:**
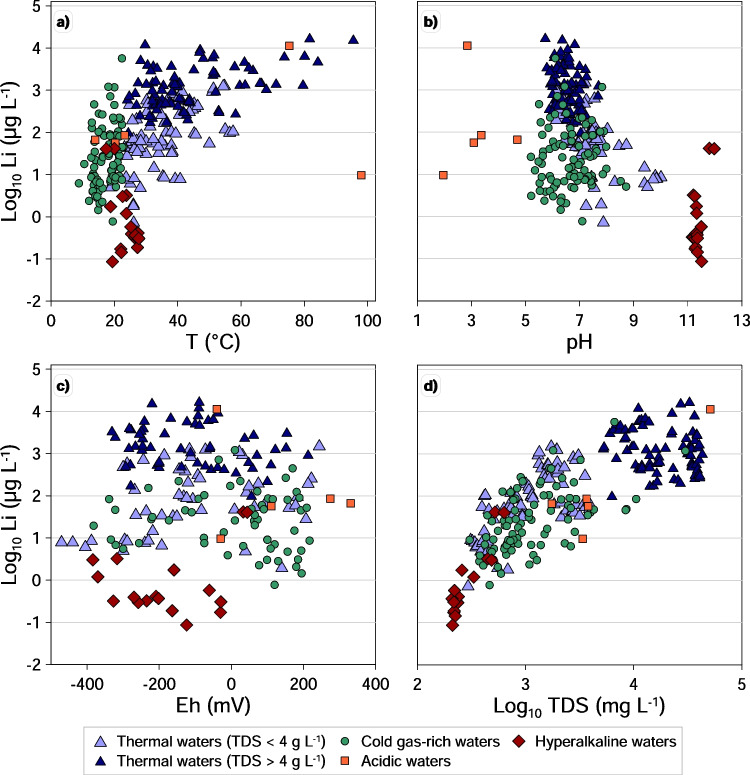
Relationships between Li concentration with the main physicochemical parameters (a) temperature; (b) pH, and (c) Eh and (d) with TDS

The solubility of various trace elements in water strongly depends on many factors like temperature, acidity, redox conditions and salinity of the solution. The relationships between the concentrations of the analysed trace elements and the main physical–chemical parameters have been explored through binary graphs found in the Supplementary Material section (Fig. S1). The concentrations of Li vs. respectively T, pH, Eh and TDS are shown in Fig. 4 as an example.

Most elements show a positive relationship with temperature. The best correlation is shown by alkalis (Li, Rb, Cs) and by Ti, Sr, As and Tl. Most of the other elements (B, V, Cr, Mn, Fe, Co, Ni, Zn, Mo, Sb, Ba and Pb) show a wide range of concentrations at low temperatures, while at high temperatures, they present only high concentrations, though sometimes not the highest. These elements are mostly influenced by the increase in water–rock interaction induced by higher temperatures. On the contrary, some elements (Be, Al, Cu, Se and Cd) show no clear relationship with temperature or even an inverse relationship (U). Although in some cases the lack of relationship may be due to the high proportion of missing values, in other cases, elements may be incorporated in solid phases that become oversaturated at higher temperatures.

Many alkaline and earth-alkaline elements and transitional metals (Fig. S1) show an inverse relationship with pH. For most of the investigated waters, pH is generally controlled by the amount of dissolved CO_2_ present in it (Li Vigni et al. 2022a). Geogenically derived CO_2_ is found in a wide range of concentrations in the sampled waters, often forming a separate free gas phase (Daskalopoulou et al. 2018). But even for most of the CO_2_-rich samples, pH does not generally reach values below 5.5. Lower values are due to the oxidation of hydrothermal origin H_2_S, which produces sulfuric acid whose dissolution is responsible for values as low as 1.96 (Li Vigni et al. 2022a). The abundance of H^+^ ions promotes the dissolution of the aquifer’s rock minerals releasing major and trace elements into the solution. However, elemental solubility may be limited by the formation of secondary minerals. This is the case, for example, of chalcophile elements (Cu, As, Se, Mo, Cd, Sb, Ba, Tl and Pb), which show generally lower concentrations in acidic waters (pH < 5). This is because these waters are very rich in sulfide and sulfate ions that form with these elements’ low solubility solid phases.

No direct or inverse relationship between Eh and elements’ concentrations can be recognised.

### Seawater contamination

Most elements show a positive relationship with salinity (TDS) (Fig. S1). Salinity in natural waters may derive from different sources (seawater, water–rock interaction, dissolution of evaporitic rocks, anthropogenic, etc.). In the case of Greek thermal and mineral waters, a TDS value above 4 g L^−1^ is almost always caused by a significant or even prevailing seawater contribution (Li Vigni et al. 2022a). This is not surprising since the Hellenic territory has high coastal development, and the most saline thermal and mineral waters were sampled close to the sea either on the coast of continental Greece or on one of its numerous islands. The contribution of trace elements from seawater is therefore significant. In some cases, contamination with seawater occurs in the shallow part of the hydrologic circuit close to the water outlet. However, mostly the seawater penetrates deep in the circuit, and being heated and charged in hydrothermal gases or simply dissolving large quantities of CO_2_, it is involved in water–rock interaction processes significantly changing its original composition. This can be recognised in the trace element vs. Cl binary graphs (Fig. S2), where the trace element/Cl ratios are nearly always higher than the same ratio in seawater. The ratios reported in the graphs refer to the open ocean (Bruland and Lohan 2003) and should here be considered indicative because the Mediterranean Sea, which surrounds Greece, is a land-locked semi-closed sea with high biological productivity and strong atmospheric input mainly from anthropogenic sources and Saharan dust (Migon 2005; Middag et al. 2022).

For all those elements with high residence times (> 10^5^ a) in the ocean and that show a geographically constant element/Cl ratio, such ratio in the Mediterranean Sea should not be very different from that of the open ocean. For most of these elements, called conservative-type by Bruland and Lohan (2003), the lowest measured element/Cl ratios are almost the same as that of seawater indicating a prevailing marine origin. The remaining samples, generally the majority, show element/Cl ratios up to three orders of magnitude higher than that of seawater, pointing to a strong contribution of water–rock interaction processes. Lithium, B, V, As, Rb, Sr, Sb and Cs follow this pattern (Fig. S2). Molybdenum, classified among the conservative-type elements (Bruland and Lohan 2003), shows a different pattern with Mo/Cl ratios being lower than the seawater ratio for saline thermal waters. This indicates a probable incorporation in, or adsorption on, newly formed secondary minerals within the hydrothermal systems. Uranium, which was not considered in the review of Bruland and Lohan (2003) but is also of conservative-type in seawater (Ku et al. 1977), shows a similar pattern to Mo (Fig. S2).

Elements that have shorter residence times in the ocean (< 10^5^ a) and are influenced by organic processes (nutrient-type — Bruland and Lohan 2003), localized inputs (scavenged-type — Bruland and Lohan 2003) or by both show generally very large variability in their element/Cl ratio in seawater. For these elements, our samples show generally element/Cl ratios higher than the highest ratio indicated by Bruland and Lohan (2003). Due to higher element/Cl ratios found in the Mediterranean Sea, related to a strong input from Saharan dust (Koçak et al. 2005) and sometimes also from volcanic activity (Censi et al. 2010), for some of these elements (e.g. Al, Ti, Mn, Fe), a contribution from seawater may be invoked for most of the saline waters. For the remaining elements and the lower salinity samples, the main source is water–rock interaction.

### Water–rock interaction

#### Chromium and nickel

Chromium and nickel are heavy metal elements and can accumulate reaching toxic environmental levels. Chromium has two main oxidation states, Cr(III) and Cr(VI). The trivalent form has low mobility and is considered an important element for human health. On the other hand, the hexavalent form is a highly mobile element in water, is considered highly toxic (Apollaro et al. 2019) and in great quantities is classified as a human and animal carcinogen (WHO 2012; California Environmental Protection Agency 2011).

Nickel is highly mobile under acidic and oxidising conditions and has two main oxidation states; Ni(II) and Ni(III). Similar to Cr, Ni can be toxic in elevated quantities (Das et al. 2019).

Both elements are strongly enriched in ultramafic and mafic rocks, as well as in their resulting sedimentary rocks. Indeed, they are partitioned into olivine and pyroxene minerals due to their compatible behaviour during crystallization from basaltic magmas. Furthermore, elevated concentrations can be related to strongly weathered environments, such as laterite and bauxite deposit.

In Greece, Cr and Ni in stream water, stream sediments and soils have been considered to be related to the Pindos and Vardar ophiolite belts, although their high concentration may also be associated with bauxite and polymetallic sulfide mineralisation (Salminen et al. 2005; De Vos et al. 2006). High concentrations of both elements were found also in groundwater in many parts of the country (Bompoti et al. 2015; Economou-Eliopoulos 2003; Kelepertzis et al. 2013; Megremi 2010; Vasileiou et al. 2019).

In the present study, high Cr and Ni concentrations (often exceeding the EU limit for drinking waters (Council Directive 2020) of 50 and 20 µg L^−1^) are located in northern and central Greece, but elevated values are found also along the SAAVA (Fig. S3a,b). This spatial distribution was compared with spatial distribution maps of stream water and stream sediment made by FOREGS (Salminen et al. 2005; De Vos et al. 2006), which show a similar pattern. Such concentrations may be of natural origin with the same sources (ultramafic rocks) evidenced by the studies of FOREGS. However, particularly for central Greece and Euboea, the impact of industrial activity (treated waste is dumped into Asopos River waters), the contaminated irrigation water and the main motorway should be taken into consideration. Moreover, the high values of these two elements in Florina basin were attributed by D’Alessandro et al. (2011), besides weathering of aquifer’s rocks, to the corrosion of the steel of the pumps used to lift the water, due to the very low pH values of the CO_2_-rich groundwater of the area.

#### Manganese and iron

Manganese is a common lithophile element, whose distribution is strongly influenced by pH-Eh conditions. It has three oxidation states: Mn(II), Mn(III) and Mn(IV). Under anoxic conditions, Mn(II) is soluble, whilst under oxidising conditions, Mn(III) and Mn(IV) ions form insoluble hydrous oxides (Hylander et al. 2000).

Iron has similar chemical behaviour to Mn, and this is the reason why these elements often exhibit a strong correlation. It has two main oxidation states, Fe(II) and Fe(III). The former is soluble under acid and reducing conditions, whereas the latter forms hydroxide due to its very low solubility (Hylander et al. 2000). The presence of elevated concentrations of H_2_S in groundwater may decrease the solubility of iron, promoting the precipitation of Fe-sulphides while CO_2_ lowering the pH may stabilise Fe(II) promoting its solubility (Salminen et al. 2005; De Vos et al. 2006).

High concentrations of Mn and Fe may be toxic to human health or at least undesirable; their concentrations in groundwater are regulated by European Directive for drinking waters (Council Directive 2020), which set a threshold of 50 µg L^−1^ for Mn and 200 µg L^−1^ for Fe. Manganese is partitioned in ferromagnesian silicates and enriched in ultramafic and mafic rocks. Indeed, in association with compatible elements, as well as Ni and Cr, elevated Mn content is indicative of mafic rocks’ weathering. On the other hand, Fe is present in several common minerals and rock-forming minerals; it is partitioned in mid-stage fractionates during magmatic processes (Salminen et al. 2005; De Vos et al. 2006).

High contents of Mn and Fe were found in northern Greece and Chios Island (Fig. S3c,d) and were associated with ophiolite belts, Fe–Ni mineralization and bauxite deposits (Salminen et al. 2005; De Vos et al. 2006). The elevated values found in Florina (North Greece) and Sperchios Basins (Central Greece) may be related to the presence of high levels of CO_2_ within the aquifers (Fig. S3c,d), which drives the dissolution of Fe–Mn-bearing minerals (D’Alessandro et al. 2011; 2014). Along SAAVA (Fig. S3c,d), the elevated concentrations of these elements may be related to CO_2_-rich and reducing hydrothermal fluids (Li Vigni et al. 2022a).

#### Arsenic and antimony

Arsenic and antimony are both metalloids and present similar chemical and toxicological properties (Elinder and Friberg 1986; Luckey et al. 1975); e.g. they form oxyanions in water (Wilson et al. 2004). Arsenic is considered a harmful element, and its natural distribution is a global concern (Ravenscroft et al. 2009). In natural waters, As has two main oxidation states: As(III) and As(V), whose distribution is strongly dependent on redox and pH conditions (Cullen and Reimer 1989), e.g. under reducing conditions, the trivalent form is predominated. Antimony may occur as Sb(III) and Sb(V) compounds and is poisonous and carcinogenic (Beyersmann and Hartwig 2008). Arsenic and antimony are naturally enriched in many hydrothermal fluids and in sulfide ore mineralization. These elements are usually used as pathfinders of epithermal and mesothermal gold deposits (Boyle 1974; Salminen et al. 2005; De Vos et al. 2006).

According to Gamaletsos et al. (2013), the main As sources in Greece are active and abandoned Pb–Zn–Ag–Au (e.g. Chalkidiki, Thrace, Attica) and laterite-bauxite mines (Euboea, Parnassos-Ghiona); lignite deposits (Florina, Drama, Ioannina); metamorphic rocks of the Attico-Cycladic Crystalline Complex; geothermal fluids related to active tectonic (Axios basin and western Chalkidiki) or recent volcanic activity (SAAVA). The As levels that people are exposed to in Greece are often higher than 10 μg L^−1^ (Christodoulidou et al. 2012), and therefore, Greece is considered an As-contaminated “hot spot” (Kampouroglou and Economou-Eliopoulos 2016).

In the present study, elevated arsenic concentrations, up to 1820 µg L^−1^ were found in northern (Chalkidiki and Thrace) and central Greece (Sperchios Basin and Euboea Island), along the SAAVA, and in Lesbos and Chios Islands, confirming the above-mentioned spatial distribution (Fig. S3e). Arsenic-enriched waters in Lesbos and Chios Islands can be related to adjacent ophiolitic formations, in which As-bearing minerals are associated (Aloupi et al. 2009; Gamaletsos et al. 2013). In the area of Chalkidiki, the elevated As levels in the groundwater are due to contamination by hydrothermal fluids (Casentini et al. 2011). Along the SAAVA, e.g. Milos, the As enrichment in water is due to the hydrothermal fluids related to recent volcanic activity. The hydrothermal fluids, along their pathway, interact with the greenschist facies of the metamorphic basement, likely the As source, enriching the vapour phase of hydrothermal fluid (Price et al. 2013). Moreover, in the southern part of Milos Island, the elevated As content is discharged by a marine shallow-water hydrothermal system enriched in gaseous As (Glasby et al. 2005).

Like As, also Sb shows elevated concentrations in northern Greece and the SAAVA (Fig. S3f). In northern Greece, Sb is present in stibnite and in sulfosalts and in the porphyry-, epithermal- and intrusion-related systems that are found in the Rhodope Massif (Tsirambides and Filippidis 2019; Tzamos et al. 2020).

#### Barium

Barium is a lithophile element and is showing a chemical behaviour similar to Ca and Sr. It is distributed in K-feldspars and micas, where it substitutes for K^+^, while in non-silicate minerals i.e. apatite and calcite, Ba^2+^ ions, substitutes for Ca^2+^ (Salminen et al. 2005; De Vos et al. 2006).

Barium concentration in natural waters is controlled by the solubility of barite (BaSO_4_) and, under alkaline conditions, by witherite (BaCO_3_). Moreover, the dispersal can be controlled by adsorption processes driven by hydrous Mn- and Fe-oxides (Salminen et al. 2005; De Vos et al. 2006).

Elevated Ba concentrations are found in Greece in the argentiferous lead deposits on the Aegean Islands of Milos, Mykonos, and Kimolos (Fig. S3g) (Siotis 1936; Wollack 1951).

#### Lead

Lead is a chalcophile metal, which is rarely found in elemental state in nature but is present in some economically important minerals like galena, anglesite and cerussite. It has two oxidation states, Pb(II) and Pb(IV), with the former being more common than the latter. It can be formed naturally from the radioactive decay of U and Th. Lead is more enriched in felsic rocks with respect to mafic and has enhanced mobility in the late stages of the magmatic processes (Macdonald et al. 1973). High Pb levels can also occur naturally due to anthropogenic sources (e.g. in gasoline, vehicle exhausts, weathering of paint on houses) (Haar 1975; Botsou et al. 2022).

In Greece, elevated Pb quantities are found in the north and north-eastern regions and along the SAAVA (Fig. S3h). High Pb contents are often related to U-rich granitic intrusions. Lead deposits (often associated with Zn) occur in Euboea, Almopia, Serres, Palea Kavala, Xanthi, Rhodope and in the north and east Aegean Sea (Arvanitidis and Constantinides 1989; Triantafyllidis et al. 2007; Vavelidis et al. 1989).

#### Uranium

Uranium is a lithophile metallic element present in several minerals. Due to its incompatible behaviour, it is enriched in late-stage differentiated magmas, such as granites, pegmatites and rhyolites (Salminen et al. 2005; De Vos et al. 2006). It is an unusual metal in water; it is more soluble under oxidative conditions, occurring in hexavalent form, which is considered to be of higher toxicity (Bone et al. 2017).

Elevated uranium levels were found in northern and central (Sperchios Basin and Euboea Island) Greece and along the SAAVA (Fig. S3i), with the highest value of 34.4 µg L^−1^ located at Picrolimni (Central Macedonia). The spatial distribution of U can be related to uraniferous granites and felsic volcanism, particularly in northern Greece. In Chalkidiki, the presence of U in water can be related to the deep circulation of geothermal waters rich in carbonate species, which regulates the formation of anionic uranyl carbonate complexes, maintaining U in soluble form (Kazakis et al. 2022; Katsoyiannis et al. 2007). Although in Chalkidiki geogenic U source is prevailing, some anthropogenic contribution may derive from fertilizers (Kazakis et al. 2022). In Florina Basin (NW Greece), the elevated U values are the result of the reaction between the upper tectonically fractured parts of the Florina granite and the magmatic or meteoric-derived acidic hydrothermal solutions (Vekios et al. 2002). The geographical distribution of U concentration in the thermal and mineral waters of Greece resembles that of agricultural soils with the highest values found in the northern part of the nation (Négrel et al. 2018).

### Environmental impact and health issues

Thermomineral waters are considered a precious resource. As part of geothermal resources, they have been utilized since antiquity (Fytikas et al. 1999). This is true also for Greece, whose geothermal resources are long known, but at present, only a small fraction of them is actually exploited (Papachristou et al. 2021). But it is since many decades that some studies evidenced that geothermal resources are not always as environmentally friendly as they were thought to be (Ellis 1978; Arnorsson 2004; Kristmannsdóttir and Ármannsson 2003). One of the main issues is that the hot and sometimes very acidic geothermal fluids mobilize great quantities of potentially harmful elements, which are finally released to the earth’s surface (Baba and Ármannsson 2006).

Thermal water released through natural springs is expected to reach a sort of dynamic equilibrium with the surrounding environment. In such equilibrium, the scarcely mobile biota, especially microbes, will have adapted to the harsh geothermal conditions (Colman et al. 2014). Of course, these geothermal features remain a hazard to mobile species like animals including humans. On the contrary, thermal waters tapped by wells and drillings with their hot and saline fluids often rich in toxic elements, if not reinjected into the subsoil, represent serious harm to the environment (Kaya et al. 2011). Environmental and human health issues have been the complaints that led to the stopping of some geothermal plants around the world (Anderson 1991). One of the best examples can be found in Greece, where the power plant at Milos produced electric energy for a few years until it was stopped by the protests of the inhabitants complaining about the H_2_S released by the extracted geothermal fluids (Kousis 1993; Marouli and Kaldellis 2001).

As previously reported, trace elements in the Greek thermal and mineral waters reach sometimes very high concentrations. The assessment of their impact on the environment and human health is not an easy task. Nevertheless, we try to obtain some useful indications about possible human health issues.

The routes of potentially harmful elements from groundwater into the human body may be either indirect (inhalation, ingestion of food contaminated by groundwater) or direct (ingestion, skin contact). Inhalation refers to elements (Hg, Rn) or compounds (CO_2_, H_2_S, etc.) that have high vapour pressures in the temperature range of the sampled waters (6.5–98 °C). Chronic (Nikolopoulos et al. 2010) or even lethal effects (D’Alessandro and Kyriakopoulos 2013) have been ascertained also in Greece for these elements or compounds, but they were not analysed in this work. Toxic effects have been observed in foods contaminated by trace elements around the world. The best example is arsenic accumulating in rice grown with arsenic-rich water (Meharg and Rahman 2003). Arsenic accumulation in irrigated agricultural soils may indicate similar problems also in Greece (Casentini et al. 2011).

Dermal absorption is considered a negligible route with respect to ingestion for inorganic chemicals in water (USEPA 2004) and is here disregarded.

For the possible ingestion route, we compared the obtained concentrations with the drinking water standards of Greece, which generally correspond to those of the European Union (Council Directive 2020). A large part of the sampled thermal and mineral waters exceeded the drinking water standards for B (52.2%), Mn (48.2%), As (41.3%) and Fe (34.4%), while only a bit (< 10%) for Al, Cr, Ni, Se, Sb, Pb and U. Some elements, although not regulated either by the Hellenic law or the EU directive, exceed limits suggested by WHO (Ba: 4.0%) or USEPA (Be: 4.0%; Tl: 2.0%) in a few samples. Finally, 30.8% of the samples exceeded the USEPA provisional guideline value of 4000 µg L^−1^ for Sr, which is intended to protect bone health in humans, especially infants (Norman et al. 2018).

However, the excess of the above-mentioned limits does not necessarily indicate a risk for humans, because most of these waters are not used for drinking purposes. Furthermore, people only occasionally drink some of these waters, while health-imposed limits are generally intended for lifetime consumption (W.H.O. 2017). Some thermal and mineral waters are among those consumed occasionally because of either their ascertained or supposed healing properties. Some of them are even considered sacred or life-giving (*ζωοδόχος πηγή* in Greek) and are dedicated to Saints or Virgin Mary.

Even though water limits should be strictly considered only for samples from sites whose water is largely consumed for drinking purposes, the impact of the remaining thermal and mineral waters should not be disregarded. Even if not used directly for human consumption, especially the waters strongly enriched in potentially harmful elements may be the source of contamination of waters used for drinking purposes or agriculture (irrigation, farm animal feeding). Examples can be found worldwide (Arnorsson 2004; Baba and Ármannsson 2006; Concha et al. 2010; Guo et al. 2008; 2009) and also in Greece (Casentini et al. 2011; D’Alessandro et al. 2011).

## Conclusions

The nationwide prospection of Greek thermal and mineral waters evidences a very large variability in the trace element concentrations. This huge variability has been evidenced previously by Li Vigni et al. (2022a) in the major ions composition and can be attributed to the complex geology of the region, which in turn influences a variety of processes acting during hydrologic circulation. Some of these processes produce “peculiar” water types. For example, serpentinization processes in ultramafic rocks lead to the formation of hyperalkaline waters with pH > 11, while the oxidation of hydrothermal derived H_2_S results in waters with very low pH (1.96–4.70). This large range of pH values together with a great variability in water temperatures (6.5–98 °C) favoured great diversity in water composition with their salinity spanning from 0.3 to 43.2 g L^−1^. Such variability is reflected also in the large range of trace element contents that is covering many orders of magnitude (up to six for Mn, Fe and Cs). For many trace elements, the highest concentrations are related to high salinity waters. Although these waters derive most of their solutes from seawater contamination, elements like Li, B, Rb, Sr and Cs show element/Cl ratios much higher than that of the sea. The high concentrations (up to 17,600, 126,000, 9230, 80,000 and 2490 µg L^−1^ respectively) attained by these elements indicate, therefore, a substantial contribution from water–rock interaction.

Both cold and thermal CO_2_-rich waters and acidic waters display elevated concentrations of Mn (up to 15,600 μg L^−1^) and Fe (up to 218,000 μg L^−1^). In these waters, low pH and Eh values stabilise more soluble reduced species such as Mn(II) and Fe(II), favouring the mobility of these elements, whilst at the water outflow, changes in environmental conditions, due to loss in CO_2_ and dissolution of O_2_, often induce extensive precipitation of oxi-hydroxides following the oxidation of these elements.

Water temperature has a strong influence on many elements with the hottest water being enriched in silica, As, Sb, Li, B, Rb and Cs.

Hyperalkaline waters are generally strongly depleted in trace elements due to incorporation in low-solubility secondary minerals; however, they are enriched in Al (up to 421 μg L^−1^). Aluminium becomes soluble at extreme pH conditions, and therefore, highly acidic waters (pH < 5) present also enhanced concentrations (up to 100,000 μg L^−1^).

In some cases, the maximum contaminant levels (MCLs) for drinking water of many elements are strongly exceeded in the under investigation waters. Such elevated concentrations of harmful elements may create hazards to human health either via direct consumption of cold mineral waters or through the mixing of highly mineralized waters, even in small proportions, with shallow groundwater. For instance, As (MCL 10 μg L^−1^) in the sampled waters reaches concentrations up to 1820 μg L^−1^ deriving from high-temperature water–rock interaction within hydrothermal circuits.

## Supplementary Information

Below is the link to the electronic supplementary material.

Supplementary file1 (PDF 6798 KB)

Supplementary file2 (PDF 2360 KB)

Supplementary file3 (PDF 2642 KB)

## Data Availability

All data have been submitted to the EarthChem repository (Li Vigni et al. 2022b-10.26022/IEDA/112712).

## References

[CR1] Abou Seedo K, Abido MS, Salih AA, Abahussain A (2017) Assessing heavy metals accumulation in the leaves and sediments of urban mangroves (Avicennia marina (Forsk.) Vierh.) in Bahrain. Internat J Ecol 8 Article ID 3978216. 10.1155/2017/3978216

[CR2] Aiuppa A, Bellomo S, Brusca L, D’Alessandro W, Federico C (2003). Natural and anthropogenic factors affecting groundwater quality of an active volcano (Mt. Etna, Italy). Appl Geochem.

[CR3] Aiuppa A, Avino R, Brusca L, Caliro S, Chiodini G, D’Alessandro W, Favara R, Federico C, Ginevra W, Inguaggiato S, Longo M, Pecoraino G, Valenza M (2006). Mineral control of arsenic content in thermal waters from volcano-hosted hydrothermal systems: Insights from island of Ischia and Phlegrean Fields (Campanian Volcanic Province, Italy). Chem Geol.

[CR4] Aiuppa A, Hall-Spencer JM, Milazzo M, Turco G, Caliro S, Di Napoli R (2021). Volcanic CO_2_ seep geochemistry and use in understanding ocean acidification. Biogeochem.

[CR5] Aloupi M, Angelidis MO, Gavriil AM, Koulousaris M, Varnavas S (2009). Influence of geology on arsenic concentrations in ground and surface water in central Lesvos, Greece. Environ Monitor Assess.

[CR6] Anderson I (1991). Blowout blights future of Hawaii’s geothermal power. New Scientist.

[CR7] Apollaro C, Fuoco I, Brozzo G, De Rosa R (2019). Release and fate of Cr(VI) in the ophiolitic aquifers of Italy: the role of Fe(III) as a potential oxidant of Cr(III) supported by reaction path modelling. Sci Total Environ.

[CR8] Arnorsson H (2004) Environmental impact of geothermal energy utilization. In: Giere R, Stille P (eds) Energy, waste, and the environment: a geochemical perspective. Geol Soc Spec Publ London 236:297–336

[CR9] Arvanitidis N, Constantinides D (1989). Base and precious metal sulphide mineralization of the Greek Rhodope Massif. Geologica Rhodopica.

[CR10] Athanasoulis K, Vakalopoulos P, Xenakis M, Persianis D, Taktikos S (2009) Periodic monitoring of spa sources of Greece. Project Report “Integrated quantitative and qualitative study of the thermomineral waters of the country” Institute of Geological and Mineral Exploration (IGME) Athens (in Greek)

[CR11] Baba A, Ármannsson H (2006). Environmental impact of the utilization of geothermal areas. Energy Sources Part B.

[CR12] Bagnato E, Aiuppa A, Parello F, D’Alessandro W, Allard P, Calabrese S (2009). Mercury concentration, speciation and budget in volcanic aquifers: Italy and Guadeloupe (Lesser Antilles). J Volcanol Geotherm Res.

[CR13] Banks D, Reimann C, Røyset O, Skarphagen H, Sæther OM (1995). Natural concentrations of major and trace elements in some Norwegian bedrock groundwaters. Appl Geochem.

[CR14] Beyersmann D, Hartwig A (2008). Carcinogenic metal compounds: recent insight into molecular and cellular mechanisms. Arch Toxicol.

[CR15] Blowes DW, Ptacek CJ, Jambor JL, Weisener CG (2003) The geochemistry of acid mine drainage. Treatise on geochemistry. Vol. 9. In: Holland HD and Turekian KK (Eds.) pp. 612. Elsevier, pp.149–204

[CR16] Bompoti N, Chrysochoou M, Dermatas D (2015). Geochemical characterisation of Greek ophiolitic environments using statistical analysis. Environ Proces.

[CR17] Bone SE, Dynes JJ, Cliff J, Bargar JR (2017). U(IV) adsorption by natural organic matter in anoxic sediments. Proc Nation Acad Sci.

[CR18] Botsou F, Sungur A, Kelepertzis E, Kypritidou Z, Daferera O, Massas I, Argyraki A, Skordas K, Soylak M (2022). Estimating remobilization of potentially toxic elements in soil and road dust of an industrialized urban environment. Environ Monitor Assess.

[CR19] Boyle RW (1974). The use of major elemental ratios in detailed geochemical prospecting utilizing primary halos. J Geochem Explor.

[CR20] Brugnone F, D’Alessandro W, Calabrese S, Li Vigni L, Bellomo S, Brusca L, Prano V, Saiano F, Parello F (2020). A Christmas gift: signature of the 24th December 2018 eruption of Mt. Etna on the chemical composition of bulk deposition in eastern Sicily. Ital J Geosci.

[CR21] Bruland KW, Lohan MC (2003) 6.02 - controls of trace metals in seawater. In: Treatise on Geochemistry (Holland HD, Turekian KK eds), Pergamon, Vol. 6, 23–47

[CR22] Calabrese S, Aiuppa A, Allard P, Bagnato E, Bellomo S, Brusca L, D’Alessandro W, Parello F (2011). Atmospheric sources and sinks of volcanogenic elements in a basaltic volcano (Etna, Italy). Geochim Cosmochim Acta.

[CR23] Calabrese S, Randazzo L, Daskalopoulou K, Milazzo S, Scaglione S, Vizzini S, Tramati CD, D’Alessandro W, Brusca L, Bellomο S, Giufrida G, Pecoraino G, Montana G, Salerno G, Giammanco S, Caltabiano T, Parello F (2016). Mount Etna volcano (Italy) as a major “dust” point source in the Mediterranean area. Arab J Geosci.

[CR24] California Environmental Protection Agency (2011) Environmental Health Hazard Assessment, Pesticide and Environmental Toxicology Branch Office, Public health goal for hexavalent chromium in drinking water, p.80

[CR25] Casentini B, Hug SJ, Nikolaidis NP (2011). Arsenic accumulation in irrigated agricultural soils in Northern Greece. Sci Total Environ.

[CR26] Censi P, Randazzo LA, Zuddas P, Saiano F, Aricò P, Andò S (2010). Trace element behaviour in seawater during Etna's pyroclastic activity in 2001: Concurrent effects of nutrients and formation of alteration minerals. J Volcanol Geotherm Res.

[CR27] Chiodini G, Frondini F, Cardellini C, Parello F, Peruzzi L (2000). Rate of diffuse carbon dioxide Earth degassing estimated from carbon balance of regional aquifers: the case of central Apennine. Italy J Geophys Res.

[CR28] Christodoulidou M, Charalambous C, Aletrari M, Nicolaidou Kanari P, Petronda A, Ward NI (2012). Arsenic concentrations in groundwaters of Cyprus. J Hydrol.

[CR29] Colman DR, Garcia JR, Crossey LJ, Karlstrom KE, Jackson-Weaver O, Takacs-Vesbach T (2014). An analysis of geothermal and carbonic springs in the western United States sustained by deep fluid inputs. Geobiol.

[CR30] Concha G, Broberg K, Grander M, Cardozo A, Palm B, Vahter M (2010). High-level exposure to lithium, boron, cesium, and arsenic via drinking water in the Andes of northern Argentina. Environ Sci Technol.

[CR31] Council Directive (2020) 20/2184/EC of 16 December 2020 on the quality of water intended for human consumption. Official Journal of the European Union, L 435, 23.12.2020, p. 1–62

[CR32] Cullen WR, Reimer KJ (1989). Arsenic speciation in the environment. Chem Rev.

[CR33] D’Alessandro W, Kyriakopoulos K (2013). Preliminary gas hazard evaluation in Greece. Nat Hazards.

[CR34] D’Alessandro W, Brusca L, Kyriakopoulos K, Bellomo S, Calabrese S (2014). A geochemical traverse along the “Sperchios Basin - Evoikos Gulf” Graben (Central Greece): origin and evolution of the emitted fluids. Mar Petrol Geol.

[CR35] D’Alessandro W, Bellomo S, Brusca L, Kyriakopoulos K, Calabrese S, Daskalopoulou K (2017). The impact of natural and anthropogenic factors on groundwater quality in an active volcanic/geothermal system under semi-arid climatic conditions: the case study of Methana peninsula (Greece). J Geochem Explor.

[CR36] D’Alessandro W, Daskalopoulou K, Calabrese S, Bellomo S (2018). Water chemistry and abiogenic methane content of a hyperalkaline spring related to serpentinization in the Argolida ophiolite (Ermioni, Greece). Mar Petrol Geol.

[CR37] D’Alessandro W, Calabrese S, Bellomo S, Brusca L, Daskalopoulou K, Li Vigni L, Randazzo L, Kyriakopoulos K (2020). Impact of hydrothermal alteration processes on element mobility and potential environmental implications at the Sousaki solfataric field (Corinthia - Greece). J Volcanol Geotherm Res.

[CR38] D’Alessandro W, Bellomo S, Brusca L, Karakazanis S, Kyriakopoulos K, Liotta M (2011) The impact on water quality of the high carbon dioxide contents of the groundwater in the area of Florina (N. Greece). In: Lambrakis N, Stournaras G, Katsanou K (eds) Advances in the Research of Aquatic Environment. Springer, Berlin, vol. 2, 135–143. 10.1007/978-3-642-24076-8_16

[CR39] Das KJ, Chandramouli Reddy R, Bagoji IB, Das S, Bagali S, Mullur L, Khodnapur JP, Biradar MS (2019). Primary concept of nickel toxicity – an overview. J Basic Clinic Physiol Pharmacol.

[CR40] Daskalopoulou K, Calabrese S, Grassa F, Kyriakopoulos K, Parello F, Tassi F, D’Alessandro W (2018). Origin of methane and light hydrocarbons in natural fluid emissions: a key study from Greece. Chem Geol.

[CR41] Daskalopoulou K, Calabrese S, Gagliano AL, D’Alessandro W (2019). Estimation of the geogenic carbon degassing of Greece. Appl Geochem.

[CR42] Daskalopoulou K, D’Alessandro W, Longo M, Pecoraino G, Calabrese S (2022). Shallow sea gas manifestations in the Aegean Sea (Greece) as natural analogues to study ocean acidification: first catalogue and geochemical characterization. Front Mar Sci.

[CR43] de Caritat P, Kofoed JE (1995) Kola Ecogeochemistry: an environmental investigation in the Arctic Europe. https://www.ngu.no/Kola/ (accessed on September 24, 2022)

[CR44] De Vos W, Tarvainen T (Chief eds), Salminen R, Reeder S, De Vivo B, Demetriades A, Pirc S, Batista MJ, Marsina K, Ottesen R-T, O'Connor PJ, Bidovec M, Lima A, Siewers U, Smith B, Taylor H, Shaw R, Salpeteur I, Gregorauskiene V, Halamic J, Slaninka I, Lax K, Gravesen P, Birke M, Breward N, Ander EL, Jordan G, Duris M, Klein P, Locutura J, Bellan A, Pasieczna A, Lis J, Mazreku A, Gilucis A, Heitzmann P, Klaver G, Petersell V (2006) Geochemical Atlas of Europe. Part 2 - interpretation of geochemical maps, additional tables, figures, maps, and related publications. Geological Survey of Finland, ISBN: 951–690–960–4

[CR45] Dotsika E, Poutoukis D, Michelot JL, Kloppmann W (2006). Stable isotope and chloride, boron study for tracing sources of boron contamination in groundwater: boron contents in fresh and thermal water in different areas in Greece. Wat Air Soil Pollut.

[CR46] Economou-Eliopoulos M (2003). Apatite and Mn, Zn, Co-enriched chromite in Ni-laterites of northern Greece and their genetic significance. J Geochem Explor.

[CR47] Elderfield H, Shultz A (1996). Mid-ocean ridge hydrothermal fluxes and the chemical composition of the ocean. Annu Rev Earth Planet Sci.

[CR48] Elinder CG, Friberg L, Friberg L, Nordberg GF, Vouk VB (1986). Antimony. Handbook on the toxicology of metals.

[CR49] Eliopoulos DG, Economou-Eliopoulos M, Economou G, Skounakis V (2020). Mineralogical and geochemical constraints on the origin of Mafic-Ultramafic-Hosted Sulphides: the Pindos Ophiolite Complex. Minerals.

[CR50] Ellis AJ (1978). Geothermal fluid chemistry and human health. Geothermics.

[CR51] Fordyce FM (2013) Selenium Deficiency and Toxicity in the Environment. In: Selinus O (ed) Essentials of Medical Geology. Springer, Dordrecht. 10.1007/978-94-007-4375-5_16

[CR52] Fytikas M, Kolios N, Cermak V, Rybach L (1979). Preliminary heat flow map of Greece. Terrestrial heat flow in Europe.

[CR53] Fytikas M, Innocenti F, Manetti P, Mazuoli R, Peccerilo A, Villari L (1984) Tertiary to Quaternary evolution of the volcanism in the Aegean Sea. In: Dixon JE, Robertson AHF (eds) The geological evolution of the Eastern Mediterranean, Geol Soc London Spec Pub 17:687–699

[CR54] Fytikas M, Margomenou-Leonidopoulou G, Cataldi R (1999) Geothermal energy in Ancient Greece: from mythology to late antiquity. In: Stories from a Heated Earth. Geothermal Resources Council and IGA, California

[CR55] Fytikas M (1977) Geological and geothermal study of Milos island. (in Greek with English abstract). Unpublished report, IGME Athens, Greece, XVII, No1

[CR56] Gagliano AL, Calabrese S, Daskalopoulou K, Cabassi J, Capecchiacci F, Tassi F, Bellomo S, Brusca L, Bonsignore M, Milazzo S, Giudice G, Li Vigni L, Parello F, D’Alessandro W (2019). Degassing and cycling of mercury at Nisyros Volcano (Greece). Geofluids.

[CR57] Gaillardet J, Viers J, Duprè B (2003) Trace elements in river waters. In: Drever JI (Ed) Treatise on Geochemistry 5: 225–272, Elsevier

[CR58] Gamaletsos P, Godelitsas A, Dotsika E, Tzamos E, Göttlicher J, Filippidis A (2013) Geological sources of As in the environment of Greece: a review. In: Scozzari A, Dotsika E (Eds) Threats to the quality of groundwater resources: prevention and control, Hdb Env Chem 40:77–113

[CR59] Geibert W (2018). Processes that regulate trace element distribution in the ocean. Elements.

[CR60] Glasby GP, Papavassiliou C, Mitsis J, Valsami-Jones E, Liakopoulos A, Rener R (2005). The Vani manganese deposit, Milos island, Greece: a fossil stratabound Mn-Ba-Pb-Zn-As-Sb-W-rich hydrothermal deposit. Develop Volcanol.

[CR61] Gomes MP, Carneiro MMLC, Garcia QS (2014) Trace elements tolerance modulated by antioxidant system in plants. In: Oxidative damage to plants. Antioxidant Networks and Signaling:523–540 10.1016/b978-0-12-799963-0.00017-4

[CR62] Guo Q, Wang Y, Liu W (2008). B, As, and F contamination of river water due to wastewater discharge of the Yangbajing geothermal power plant, Tibet, China. Environ Geol.

[CR63] Guo Q, Wang Y, Liu W (2009). Hydrogeochemistry and environmental impact of geothermal waters from Yangyi of Tibet, China. J Volcanol Geotherm Res.

[CR64] Haar GT (1975). Lead in the environment – origins, pathways, and sinks. Environ Qual Saf Suppl.

[CR65] Hahn A, Naden J, Treloar PJ, Kilias SP, Rankin AH, Forward P (2012) A new timeframe for the mineralization in the Kassandra mine district, N. Greece: deposit formation during metamorphic core complex exhumation. European Mineralogical Conference 2012 Vol. 1, EMC2012–742

[CR66] Hylander L, Meili M, Oliveira L, Silva E, Guimarães J-R, Araujo D, Neves R, Stachiw R, Barros A, Silva G (2000). Relationship of mercury with aluminum, iron, and manganese oxy-hydroxides in sediments from the Alto Pantanal, Brazil. Sci Total Environ.

[CR67] Jahan S, Strezov V (2019). Assessment of trace elements pollution in the sea ports of New South Wales (NSW), Australia using oysters as bioindicators. Sci Rep.

[CR68] Jolivet L, Lecomte E, Huet B, Denèle Y, Lacombe O, Labrousse L, Le Pourhiet L, Mehl C (2010). The North Cycladic Detachment System. Earth Planet Sci Lett.

[CR69] Kabata-Pendias A (2010). Trace elements in soils and plants.

[CR70] Kampouroglou EE, Economou-Eliopoulos M (2016). Assessment of the environmental impact by As and heavy metals in lacustrine travertine limestone and soil in Attica, Greece: mapping of potentially contaminated sites. CATENA.

[CR71] Kaprara E, Kazakis N, Simeonidis K, Coles S, Zouboulis AI, Samaras P, Mitrakas M (2015). Occurrence of Cr(VI) in drinking water of Greece and relation to the geological background. J Hazard Mater.

[CR72] Katsanou K, Siavalas G, Lambrakis N (2012). The thermal and mineral springs of Aitoloakarnania Prefecture: function mechanism and origin of groundwater. Environ Earth Sci.

[CR73] Katsanou K, Siavalas G, Panagopoulos G, Lambrakis N (2022) Rare earth element patterns in a rapidly changing karst environment. Appl Geoc 146:105462. 10.1016/j.apgeochem.2022.105462

[CR74] Katsoyiannis IA, Hug SJ, Ammann A, Zikoudi A, Hatziliontos C (2007). Arsenic speciation and uranium concentrations in drinking water suplly wells in Northern Greece: correlations with redox indicative parameters and implications for groundwater treatment. Sci Total Environ.

[CR75] Kaya E, Zarrouk SJ, O'Sullivan MJ (2011). Reinjection in geothermal fields: a review of worldwide experience. Renew Sustain En Rev.

[CR76] Kazakis N, Busico G, Ntona MM, Philippou K, Kaprara E, Mitrakas M, Bannenberg M, Ioannidou A, Pashalidis I, Colombani N, Mastrocicco M, Voudouris K (2022). The origin of uranium in groundwater pf the eastern Halkidiki region, northern Greece. Sci Total Environ.

[CR77] Kelepertsis A, Tziritis E, Kelepertsis E, Leontakianakos G, Pallas K (2009). Hydrogeochemical characteristics and genetic implications of Edipsos thermal springs, north Euboea, Greece. Centr Eur J Geosc.

[CR78] Kelepertzis E, Galanos E, Mitsis I (2013). Origin, mineral speciation and geochemical baseline mapping of Ni and Cr in agricultural topsoils of Thiva Valley (central Greece). J Geochem Explor.

[CR79] Kilias SP, Naden J, Cheliotis I, Sheperd TJ, Constandinidou H, Crossing J, Simos I (2001). Epithermal gold mineralisation in the active Aegean Volcanic Arc: the Profitis Ilias deposit, Milos Island, Greece. Miner Depos.

[CR80] Koçak M, Kubilay N, Herut B, Nimmo M (2005). Dry atmospheric fluxes of trace metals (Al, Fe, Mn, Pb, Cd, Zn, Cu) over the levantine basin: a refined assessment. Atmos Environ.

[CR81] Koller M, Saleh HM, Saleh HEM, El-Adham E (2018). Introductory chapter: an introduction to trace elements. Trace elements - human health and environment.

[CR82] Kouras A, Katsoyiannis I, Voutsa D (2007). Distribution of arsenic in groundwater in the area of Chalkidiki, Northern Greece. J Hazard Mater.

[CR83] Kousis M (1993). Collective resistance and sustainable development in rural Greece: the case of geothermal energy on the island of Milos. Sociol Rural.

[CR84] Kristmannsdóttir H, Ármannsson H (2003). Environmental aspects of geothermal energy utilization. Geothermics.

[CR85] Ku T-L, Mathieu GG, Knauss KG (1977). Uranium in open ocean: concentration and isotopic composition. Deep Sea Res.

[CR86] Lambrakis N, Kallergis G (2005). Contribution to the study of Greek thermal springs: hydrogeological and hydrochemical characterized and origin of the thermal waters. Hydrogeol J.

[CR87] Lambrakis N, Stamatis GN (2008). Contribution to the study of thermal waters in Greece: chemical patterns and origin of thermal water in the thermal springs of Lesvos. Hydrol Process.

[CR88] Lambrakis N, Zagana E, Katsanou K (2013). Geochemical patterns and origin of alkaline thermal waters in Central Greece (Platystomo and Smokovo areas). Environ Earth Sci.

[CR89] Lambrakis N, Katsanou K, Siavalas G (2013a) Geothermal fields and thermal waters of Greece: an overview. In: Baba A, Bundschuh J, Chandrasekaram D (eds) Geothermal systems and energy resources. Turkey and Greece, Sustainable Energy Developments 7, CRC Press, 25–46

[CR90] Landerer X (1843). Beschreibung der heilquellen Griechenlands.

[CR91] Le Pichon X, Şengör AMC, Demirbağ E, Rangin C, İmren C, Armijo R, Gӧrür N, Ҫağatay N, Mercier de Lepinay B, Meyer B, Saatҫilar R, Tok B (2001). The active Main Marmara Fault. Earth Planet Sci Lett.

[CR92] Li Vigni L, Daskalopoulou K, Calabrese S, Parello F, D’Alessandro W (2021). Geochemical characterisation of the alkaline and hyperalkaline groundwater in the Othrys Ophiolite Massif, central Greece. Ital J Geosci.

[CR93] Li Vigni L, Daskalopoulou K, Calabrese S, Kyriakopoulos K, Parello F, Brugnone F, D’Alessandro W (2022). Geochemical characterisation of the thermo-mineral waters of Greece. Environ Geochem Health.

[CR94] Li Vigni L, Daskalopoulou K, Calabrese S, Kyriakopoulos K, Bellomo S, Brusca L, Brugnone F, Parello F, D'Alessandro W (2022b) Chemical-physical parameters, major, minor and trace elements composition of thermo-mineral groundwaters of Greece, Version 1.0. Interdisciplinary Earth Data Alliance (IEDA). 10.26022/IEDA/112712.

[CR95] Liotta M, Martínez Cruz M, Ferrufino A, Rüdiger J, Gutmann A, Cerda KVR, Bobrowski N, de Moor M (2021). Magmatic signature in acid rain at Masaya volcano, Nicaragua: inferences on element volatility during lava lake degassing. Chem Geol.

[CR96] Luckey TD, Venugopal B, Hutcheson D (1975) Heavy metal toxicity safety and hormology, in: Coulston F, Korte F (Eds), Environmental Quality and Safety, Supplement vol. I, Georg Thieme, Stuttgart 1057514

[CR97] Macdonald R, Upton BGJ, Thomas JE (1973). Potassium- and fluorine-rich hydrous phase coexisting with peralkaline granite in South Greenland. Earth Planet Sci Lett.

[CR98] Marouli C, Kaldellis JK (2001) Risk in the Greek electricity production sector. Proc 7th Internat Conf Environ Sci Technol Ermoupolis, Syros Island, Greece, Sept. 2001, 305–314

[CR99] McClusky S, Balassanian S, Barka A, Demir C, Ergintav S, Georgiev I, Gurkan O, Hamburger M, Hurst K, Kahle H, Kastens K, Kekelidze G, King R, Kotzev V, Lenk O, Mahmoud S, Mishin A, Nadariya M, Ouzounis A, Paradissis D, Peter Y, Prilepin M, Reilinger R, Sanli I, Seeger H, Tealeb A, Toksöz MN, Veis G (2000). Global Positioning System constraints on plate kinematics and dynamics in the Eastern Mediterranean and Caucasus. Tectonophysics.

[CR100] Megremi I (2010). Distribution and bioavailability of Cr in central Euboea. Greece Open Geosci.

[CR101] Meharg AA, Rahman MM (2003). Arsenic contamination of Bangladesh paddy field soils: implications for rice contribution to arsenic consumption. Environ Sci Technol.

[CR102] Melfos V, Voudouris PC (2012). Geological, mineralogical and geochemical aspects for critical and rare metals in Greece. Minerals.

[CR103] Mercier J-L (1981). Extensional-compressional tectonics associated with the Aegean arc: comparison with the Andean Cordillera of south Peru-north Bolivia. Philos T Roy Soc A.

[CR104] Middag R, Rolison JM, George E, Gerringa LJA, Rijkenberg MJA, Stirling CH (2022). Basin scale distributions of dissolved manganese, nickel, zinc and cadmium in the Mediterranean Sea. Mar Chem.

[CR105] Migon C (2005) Trace metals in the Mediterranean Sea. In: Saliot A (ed) The Mediterranean Sea. Handbook of Environmental Chemistry, vol 5K. Springer, Berlin, Heidelberg. 10.1007/b107146

[CR106] Mountrakis D (1985). Geology of Greece.

[CR107] Mountrakis D (2010). Geology and Geotectonic Evolution of Greece.

[CR108] Négrel P, De Vivo B, Reimann C, Ladenberger A, Cicchella D, Albanese S, Birke M, De Vos W, Dinelli E, Lima A, O'Connor PJ, Salpeteur I, Tarvainen T, the GEMAS Project Team (2018). U-Th signatures of agricultural soil at the European continental scale (GEMAS): distribution, weathering patterns and processes controlling their concentrations. Sci Total Environ.

[CR109] Nikolopoulos D, Vogiannis E, Petraki E, Zisos A, Louizi A (2010). Investigation of the exposure to radon and progeny in the thermal spas of Loutraki (Attica-Greece): results from measurements and modelling. Sci Total Environ.

[CR110] Nordstrom DK, McCleskey RB, Ball JW (2009). Sulfur geochemistry of hydrothermal waters in Yellowstone National Park: IV acid-sulfate waters. Appl Geochem.

[CR111] Norman JE, Toccalino PL, Morman SA (2018) Health-based screening levels for evaluating water-quality datA, second ed. U.S. Geol. Surv. National Water-Quality Assessment Program web page. 10.5066/F71C1TWP

[CR112] Papachristou M, Dalambakis P, Arvanitis A, Mendrinos D, Andritsos N (2021) Geothermal developments in Greece – country update 2015–2020. Proceedings of the World Geothermal Congress 2020+1, Reykjavik, Iceland, April - October 2021

[CR113] Pavlides S, Caputo R (1994). The North Aegean Sea region: a tectonic paradox?. Terra Nova.

[CR114] Pavlides S, Caputo R, Sboras S, Chatzipetros A, Papathanasiou G, Valkaniotis S (2010). The Greek catalogue of active faults and database of seismogenic sources. Bull Geol Soc Greece.

[CR115] Pearce JM (Ed) (2004) Natural analogues for the geological storage of CO_2_. Final report of the Nascent project. British Geological Survey Technical Report, 122 pp

[CR116] Pe-Piper G, Zhang Y, Piper DJW, Prelević D (2014). Relationship of Mediterranean type lamproites to large shoshonite volcanoes, Miocene of Lesbos, NE Aegean Sea. Lithos.

[CR117] Pertessis M (1937) Thermomineral Springs of Greece. Publication of the Geological Survey of Greece, n. 24. Athens (in Greek)

[CR118] Price RE, Savov I, Planer-Friedrich B, Bühring SI, Amend J, Pichler T (2013). Processes influencing extreme As enrichment in shallow-sea hydrothermal fluids of Milos Island, Greece. Chem Geol.

[CR119] Randazzo P, Caracausi A, Aiuppa A, Cardellini C, Chiodini G, D’Alessandro W, Li Vigni L, Papic P, Marinkovic G, Ionescu A (2021). Active degassing of deeply sourced fluids in central europe: new evidences from a geochemical study in Serbia. Geochem Geophys Geosys.

[CR120] Ravenscroft P, Brammer H, Richards K (2009) Arsenic pollution: a global synthesis. Wiley-Blackwell. ISBN: 978–1–405–18601–8

[CR121] Reimann C, de Caritat P (2005). Distinguishing between natural and anthropogenic sources for elements in the environment: regional geochemical surveys versus enrichment factors. Sci Total Environ.

[CR122] Reimann C, Äyräs M, Chekushin V, Bogatyrev I, Boyd R, de Caritat P, Dutter R, Finne TE, Halleraker JH, Jæger Ø, Kashulina G, Lehto O, Niskavaara H, Pavlov V, Räisänen ML, Strand T, Volden T (1998) Environmental Geochemical Atlas of the Central Barents Region. ISBN 82-7385-176-1. NGU-GTK-CKE Special Publication, Geological Survey of Norway, Trondheim, Norway, 745 pp

[CR123] Rognerud S, Fjeld E (2001). Trace element contamination of Norwegian Lake sediments. Ambio.

[CR124] Salminen R (Chief Ed), Batista M, Bidovec M, Demetriades A, De Vivo B, De Vos W, Duris M, Gilucis A, Gregorauskienė V, Halamić J, Heitzmann P, Lima A, Jordan G, Klaver G, Klein P, Lis J, Locutura J, Marsina K, Mazreku A, O'Connor PJ, Olsson SÅ, Ottesen R-T, Petersell V, Plant JA, Reeder S, Salpeteur I, Sandström H, Siewers U, Steenfelt A, Tarvainen T (2005) FOREGS Geochemical Atlas of Europe, Part 1: Background Information, Methodology and Maps. Geological Survey of Finland, ISBN: 951–690–921–3

[CR125] Siotis GJ (1936) The barytes deposits of Greece: sands, clays Miner 3:43–46

[CR126] Skarpelis N (2002). Geodynamics and evolution of the Miocene mineralization in the Cycladic-Pelagonian belt, Hellenides. Bull Geol Soc Greece.

[CR127] Stamatakis MG, Evangelos PT, Evelpidou N (2009). The geochemistry of boron-rich groundwater of the Karlovassi Basin, Samos Islands, Greece. Centr Eur J Geosci.

[CR128] Tamburello G, Pondrelli S, Chiodini G, Rouwet D (2018). Global-scale control of extensional tectonic of CO_2_ earth degassing. Nature Commun.

[CR129] Taymaz T, Jackson J, McKenzie D (1991). Active tectonics of the north and central Aegean Sea. Geophys J Internat.

[CR130] Triantafyllidis S, Skarpelis N, Komnitsas K (2007). Environmental characterization and geochemistry of Kirki, Thrace, NE Greece. Abandoned flotation tailing dumps. Environ Forensics.

[CR131] Tsirambides A, Filippidis A (2019) Sb- Bi-bearing metallogeny of the Serbomacedonian-Rhodope Metallogenetic Belt (SRMB). In: Proceedings of the Abstracts of 15th International Congress of the Geological Society of Greece, Athens, Greece, 22–24 May 2019

[CR132] Tzamos E, Filippidis A, Rassios A, Grieco G, Michailidis K, Koroneos A, Stamoulis K, Pedrotti M, Gamaletsos PN (2016). Major and minor element geochemistry of chromite from the Xerolivado-Skoumtsa mine, Southern Vourinos: implications for chrome ore exploration. J Geochem Explor.

[CR133] Tzamos E, Gamaletsos PN, Grieco G, Bussolesi M, Xenidis A, Zouboulis A, Dimitriadis D, Pontikes Y, Godelitsas A (2020). New insights into the mineralogy and geochemistry of Sb Ores from Greece. Minerals.

[CR134] Tziritis E, Kelepertzis A (2011) Trace and ultra-trace element hydrogeochemistry of Lesvos thermal springs. In: Lambrakis N, Stournaras G, Katsanou K (eds) Advances in the Research of Aquatic Environment. Springer, Berlin, vol. 2, 185–192

[CR135] U.S. Environmental Protection Agency (2004) Risk assessment guidance for superfund: volume I human health evaluation manual (Part E, supplemental guidance for dermal risk assessment) ("Part E")

[CR136] Vasileiou E, Papazotos P, Dimitrakopoulos D, Perraki M (2019). Expounding the origin of chromium in groundwater of the Sarigkiol Basin, Western Macedonia, Greece: a cohesive statistical approach and hydrochemical study. Environ Monit Assess.

[CR137] Vavelidis M, Filippidis A, Michailidis K, Evangelou E (1989). The polymetallic ore mineralization of the Kirki area, Alexandroupolis district, Northeast Greece. Geologica Rhodopica.

[CR138] Vekios P, Kelepertzis A, Bourithi I, Mparlas K (2002). The hydrothermal Na-Ca alteration at the marginal part of the Florina granite and the associated magnetite-hematite bands with thorium and uranium mineralization. Chin J Geochem.

[CR139] Wada O (2004). What are trace elements? —Their deficiency and excess states. Japan Med Ass J.

[CR140] Wang Y, Li P, Guo Q, Jiang Z, Liu M (2018). Environmental biogeochemistry of high arsenic geothermal fluids. Appl Geochem.

[CR141] Wang F (2009) Factor analysis and principal-components analysis. In: International Encyclopedia of Human Geography Kitchin R, Thrift N (eds), Elsevier 10.1016/B978-008044910-4.00434-X

[CR142] WHO (2012) International Agency for Research on Cancer, IARC monographs on the evaluation of carcinogenic risks to humans. a review of human carcinogens part C: arsenic, metals, fibres, and dusts 100:147–168 PMC4781271 23189751

[CR143] WHO (2017) Guidelines for drinking-water quality: fourth edition incorporating the first addendum. World Health Organization, 541 pp 28759192

[CR144] Wilson NJ, Craw D, Hunter K (2004). Antimony distribution and environmental mobility at an historic antimony smelter site, New Zealand. Environ Pollut.

[CR145] Wollack O (1951). Die Schwerspatlagerstatte von Kavos-Pilonisi auf der Insel Milos, Griechenland. Wien Berg- Und Hüttenmännische Monatshefte.

[CR146] Yan K, Wang C, Mischke S, Wang L, Yu X, Meng L (2021). Major and trace-element geochemistry of Late Cretaceous clastic rocks in the Jitai Basin, southeast China. Sci Rep.

[CR147] Zimmermann MB, Boelaert K (2015). Iodine deficiency and thyroid disorders. Lancet Diabetes Endocrinol.

