# Supplementary material for: Characterization of trace elements in thermal and mineral waters of Greece
Source: Environ Sci Pollut Res Int. 2023 Jun 3;30(32):78376–93. doi: 10.1007/s11356-023-27829-x (PMC10313562; doi:10.1007/s11356-023-27829-x)
Supplement: Supplementary file 1 — Supplementary file1 (PDF 6798 KB) [file 11356_2023_27829_MOESM1_ESM.pdf]

Supplementary material

Figure S1

Relationships between trace element concentrations with the main physicochemical parameters (temperature, pH, Eh) and TDS. In each figure a), b), c) and d) indicate respectively temperature, pH, Eh and TDS and the numbers indicate 1 = B, 2 = Be, 3 = Al, 4 = Ti, 5 = V, 6 = Cr, 7 = Mn, 8 = Fe, 9 = Co, 10 = Ni, 11 = Cu, 12 = Zn, 13 = As, 14 = Se, 15 = Rb, 16 = Sr, 17 = Mo, 18 = Cd, 19 = Sb, 20 = Cs, 21 = Ba, 22 = Tl, 23 = Pb and 24 = U.

Characterization of trace elements in thermal and mineral waters of Greece

Environmental Science and Pollution Research

Lorenza Li Vigni[1], Kyriaki Daskalopoulou[2,3], Sergio Calabrese[1,4], Konstantinos Kyriakopoulos[5], Sergio Bellomo[4], Lorenzo Brusca[4], Filippo Brugnone[1], Walter D'Alessandro[4*]

1) University of Palermo, DiSTeM, via Archirafi 36, Palermo, Italy
2) University of Potsdam, Institute of Geosciences, Karl-Liebknecht-Str. 24-25, Potsdam-Golm, Germany.
3) GeoForschungs Zentrum, Physics of Earthquakes and Volcanoes, Helmholtzstraße 6/7, Potsdam, Germany
4) Istituto Nazionale di Geofisica e Vulcanologia, sezione di Palermo, via Ugo La Malfa 153, Italy
5) National and Kapodistrian University of Athens, Faculty of Geology and Geoenvironment, Panestimioupolis, Ano Ilissia, Greece

corresponding author: walter.dalessandro@ingv.it

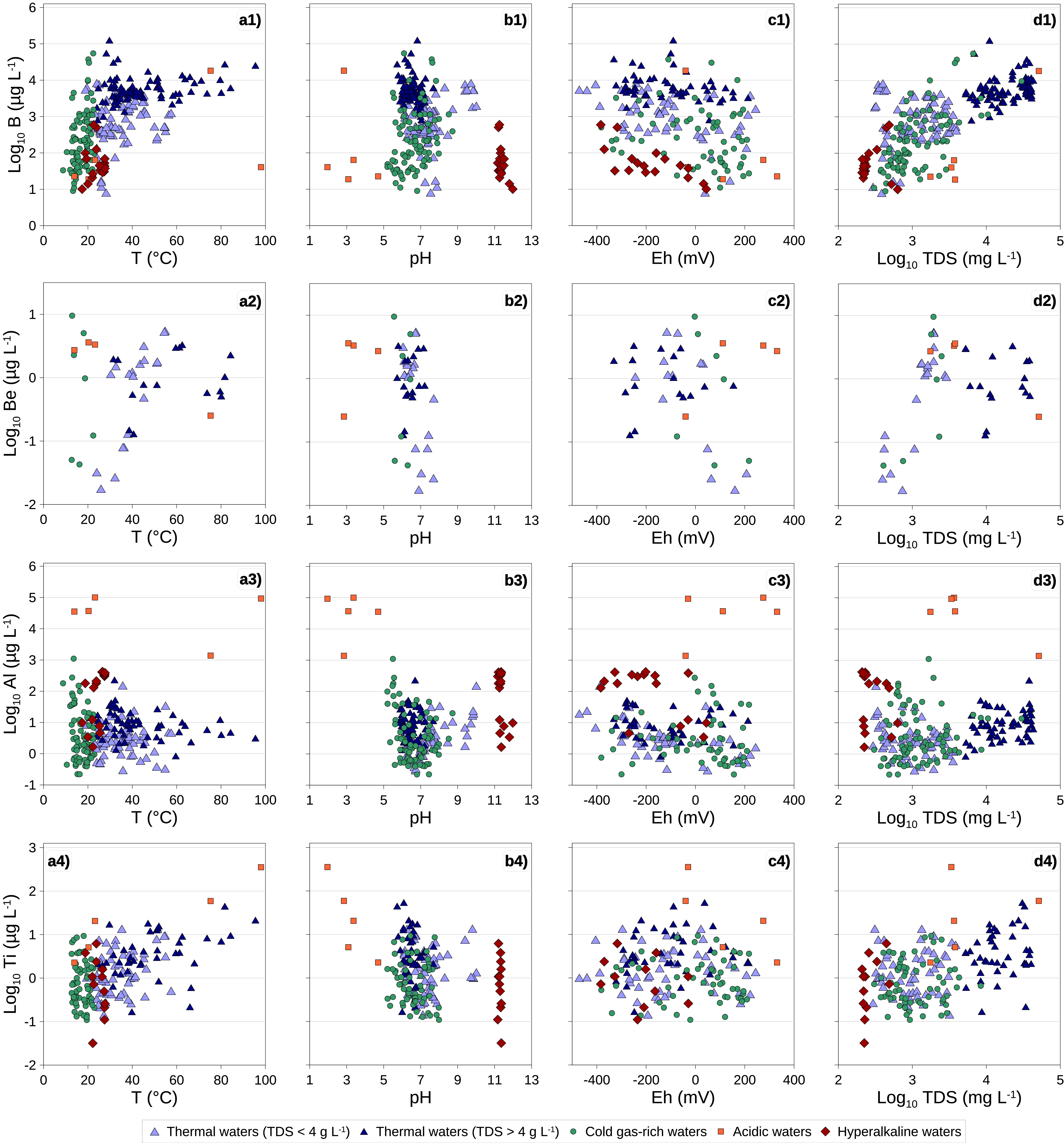
a1)
b1)
c1)
d1)
a2)
b2)
c2)
d2)
a3)
b3)
c3)
d3)
a4)
b4)
c4)
d4)
Log10 B (μg L-1)
Log10 Be (μg L-1)
Log10 Al (μg L-1)
Log10 Ti (μg L-1)
T (°C)
pH
Eh (mV)
Log10 TDS (mg L-1)
Thermal waters (TDS < 4 g L-1)
Thermal waters (TDS > 4 g L-1)
Cold gas-rich waters
Acidic waters
Hyperalkaline waters

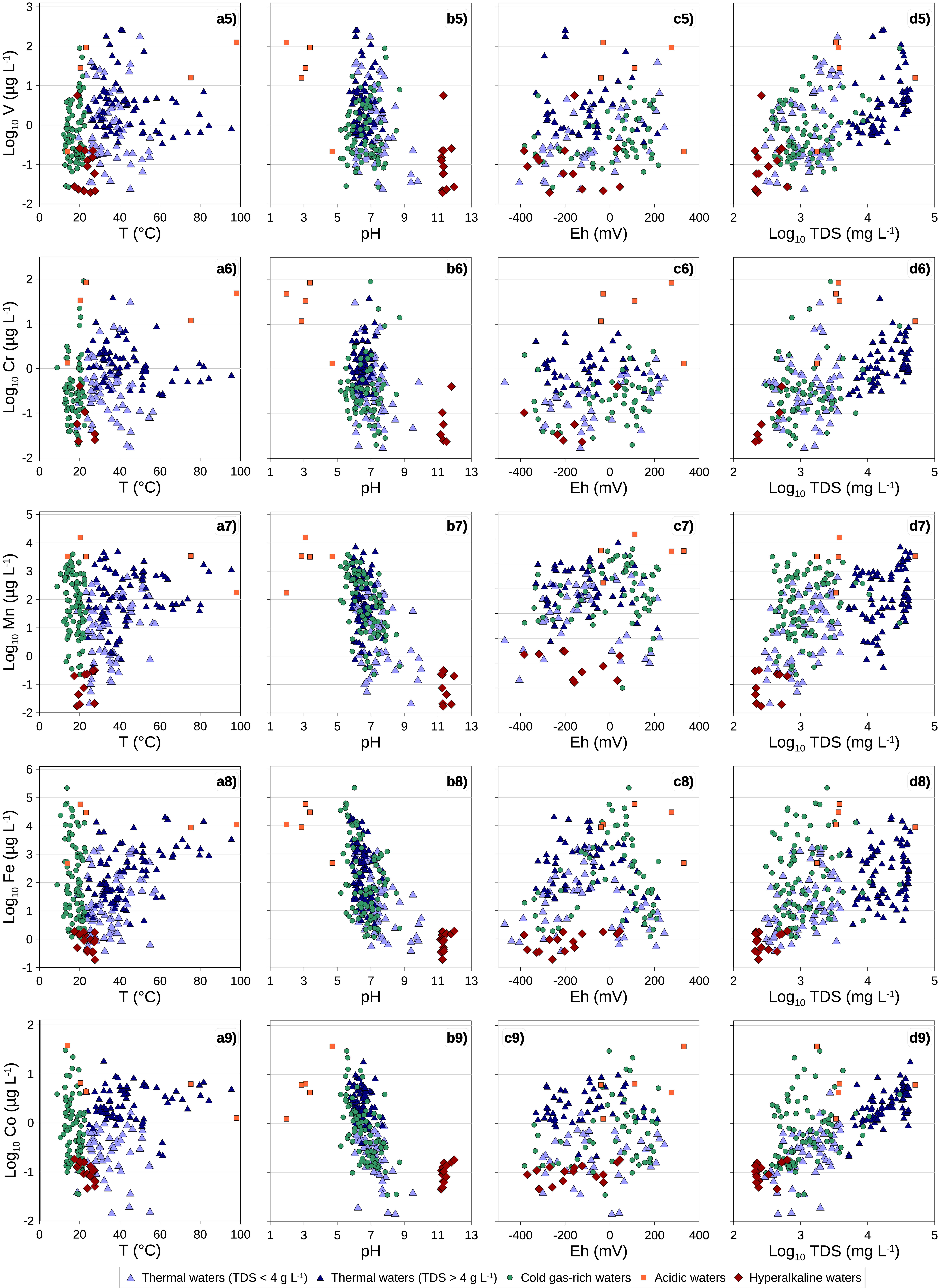

a5) b5) c5) d5)
a6) b6) c6) d6)
a7) b7) c7) d7)
a8) b8) c8) d8)
a9) b9) c9) d9)
Log10 V (µg L-1)
Log10 Cr (µg L-1)
Log10 Mn (µg L-1)
Log10 Fe (µg L-1)
Log10 Co (µg L-1)
T (°C)
pH
Eh (mV)
Log10 TDS (mg L-1)
Thermal waters (TDS < 4 g L-1)
Thermal waters (TDS > 4 g L-1)
Cold gas-rich waters
Acidic waters
Hyperalkaline waters

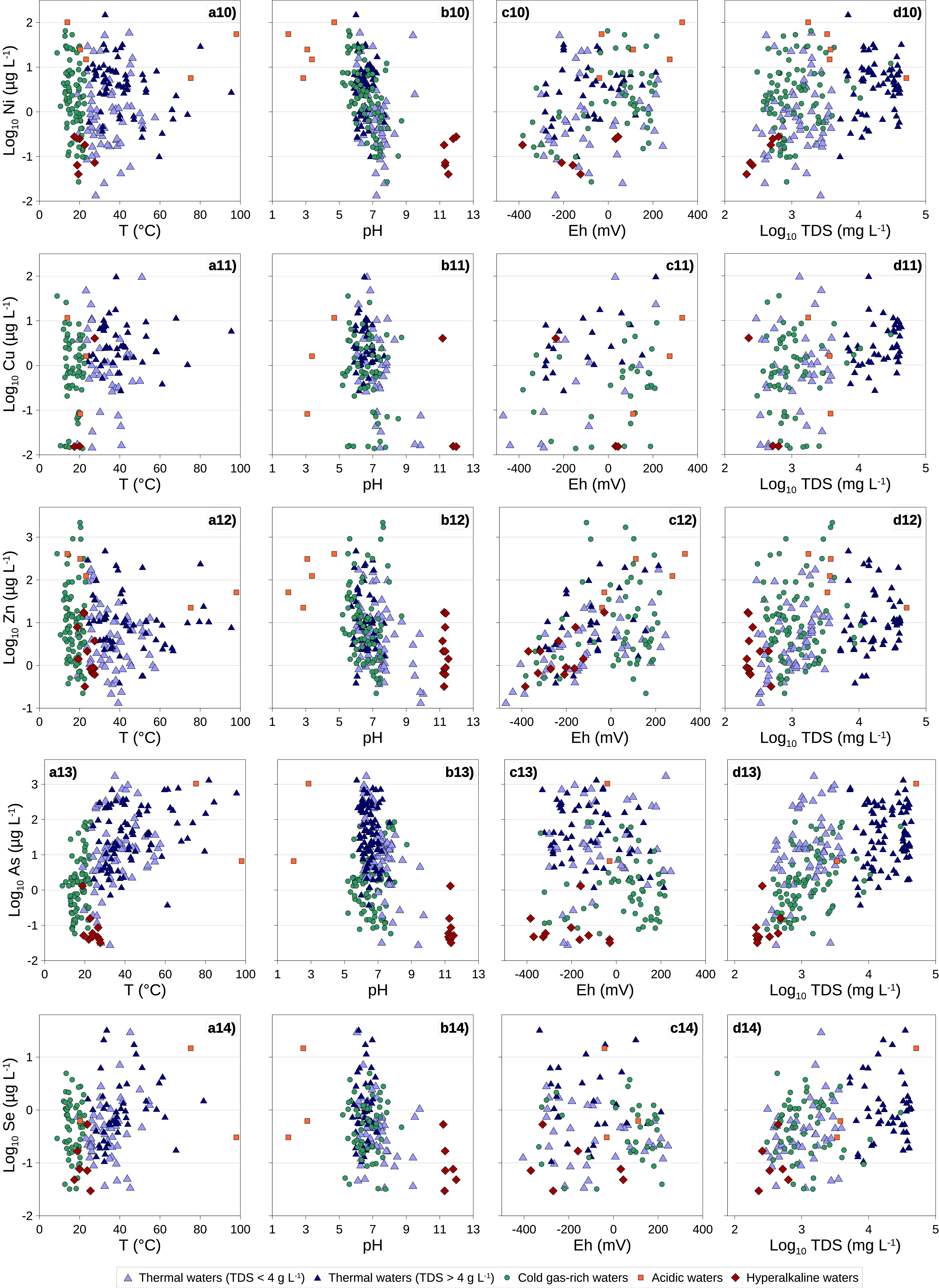

a10)
b10)
c10)
d10)
Log10 Ni (µg L-1)
a11)
b11)
c11)
d11)
Log10 Cu (µg L-1)
a12)
b12)
c12)
d12)
Log10 Zn (µg L-1)
a13)
b13)
c13)
d13)
Log10 As (µg L-1)
a14)
b14)
c14)
d14)
Log10 Se (µg L-1)
T (°C)
pH
Eh (mV)
Log10 TDS (mg L-1)
Thermal waters (TDS < 4 g L-1)
Thermal waters (TDS > 4 g L-1)
Cold gas-rich waters
Acidic waters
Hyperalkaline waters

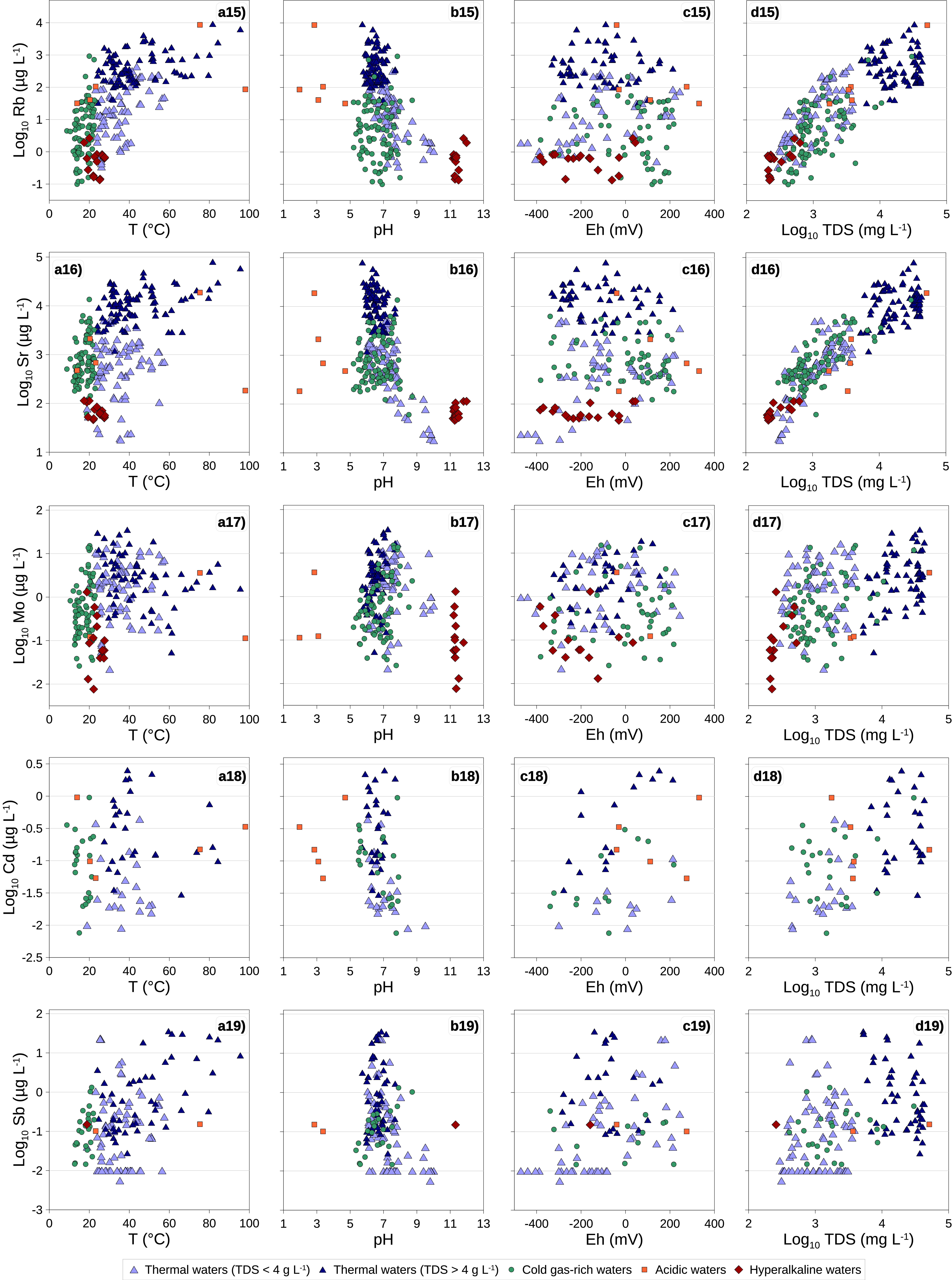

a15)
b15)
c15)
d15)
a16)
b16)
c16)
d16)
a17)
b17)
c17)
d17)
a18)
b18)
c18)
d18)
a19)
b19)
c19)
d19)
Log10 Rb (µg L-1)
Log10 Sr (µg L-1)
Log10 Mo (µg L-1)
Log10 Cd (µg L-1)
Log10 Sb (µg L-1)
T (°C)
pH
Eh (mV)
Log10 TDS (mg L-1)
Thermal waters (TDS < 4 g L-1)
Thermal waters (TDS > 4 g L-1)
Cold gas-rich waters
Acidic waters
Hyperalkaline waters

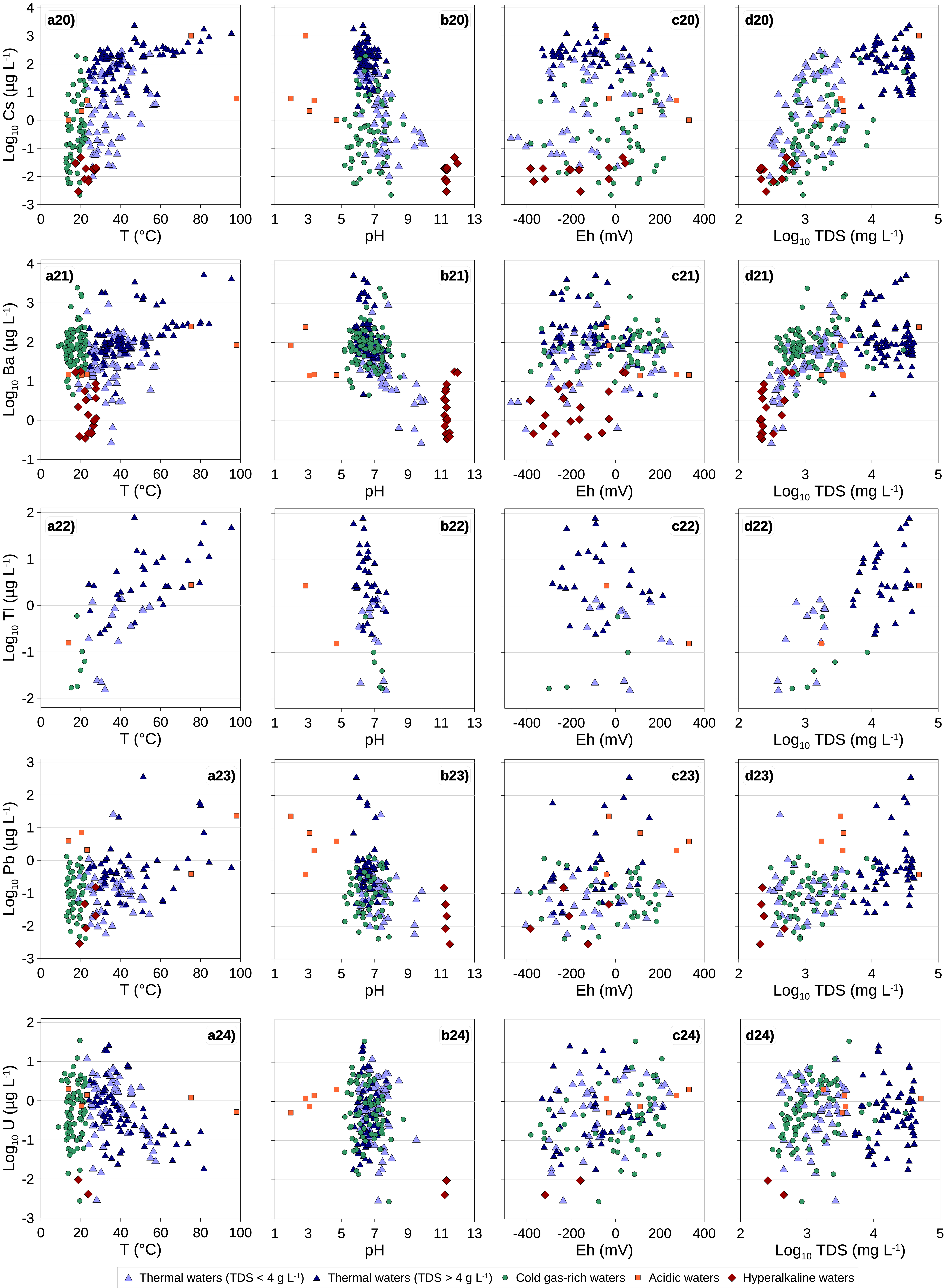

a20)
b20)
c20)
d20)
a21)
b21)
c21)
d21)
a22)
b22)
c22)
d22)
a23)
b23)
c23)
d23)
a24)
b24)
c24)
d24)
Log_{10} Cs (µg L^{-1})
Log_{10} Ba (µg L^{-1})
Log_{10} Tl (µg L^{-1})
Log_{10} Pb (µg L^{-1})
Log_{10} U (µg L^{-1})
T (°C)
pH
Eh (mV)
Log_{10} TDS (mg L^{-1})
Thermal waters (TDS < 4 g L^{-1})
Thermal waters (TDS > 4 g L^{-1})
Cold gas-rich waters
Acidic waters
Hyperalkaline waters
