# Supplementary material for: Characterization of trace elements in thermal and mineral waters of Greece
Source: Environ Sci Pollut Res Int. 2023 Jun 3;30(32):78376–93. doi: 10.1007/s11356-023-27829-x (PMC10313562; doi:10.1007/s11356-023-27829-x)
Supplement: Supplementary file 2 — Supplementary file2 (PDF 2360 KB) [file 11356_2023_27829_MOESM2_ESM.pdf]

Supplementary material

Figure S2

Relationships between trace element concentrations with chloride concentrations. a) = Li, b) = Be, c) = B, d) = Al, e) = Ti, f) = V, g) = Cr, h) = Mn, i) = Fe, j) = Co, k) = Ni, l) = Cu, m) = Zn, n) = As, o) = Se, p) = Rb, q) = Sr, r) = Mo, s) = Cd, t) = Sb, u) = Cs, v) = Ba, w) = Tl, x) = Pb and y) = U. Dashed lines are the element/Cl ratios in ocean water (Bruland and Lohan 2003). If two dashed lines are present they represent minimum and maximum element/Cl ratios in ocean water (Bruland and Lohan 2003).

Characterization of trace elements in thermal and mineral waters of Greece

Environmental Science and Pollution Research

Lorenza Li Vigni[1], Kyriaki Daskalopoulou[2,3], Sergio Calabrese[1,4], Konstantinos Kyriakopoulos[5], Sergio Bellomo[4], Lorenzo Brusca[4], Filippo Brugnone[1], Walter D'Alessandro[4*]

1) University of Palermo, DiSTeM, via Archirafi 36, Palermo, Italy
2) University of Potsdam, Institute of Geosciences, Karl-Liebknecht-Str. 24-25, Potsdam-Golm, Germany.
3) GeoForschungs Zentrum, Physics of Earthquakes and Volcanoes, Helmholtzstraße 6/7, Potsdam, Germany
4) Istituto Nazionale di Geofisica e Vulcanologia, sezione di Palermo, via Ugo La Malfa 153, Italy
5) National and Kapodistrian University of Athens, Faculty of Geology and Geoenvironment, Panestimioupolis, Ano Ilissia, Greece

corresponding author: walter.dalessandro@ingv.it

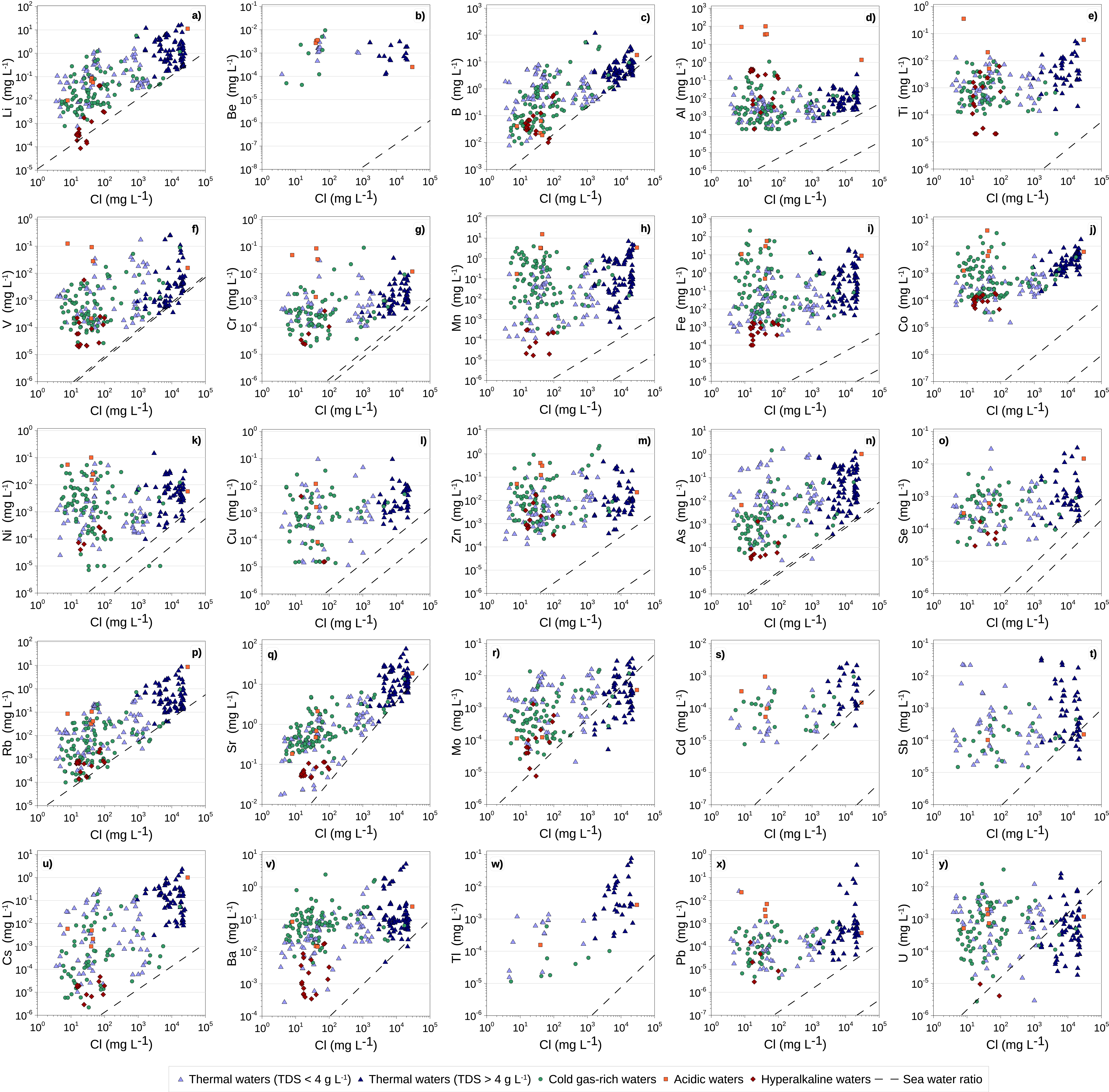

a)
Li (mg L-1)
b)
Be (mg L-1)
c)
B (mg L-1)
d)
Al (mg L-1)
e)
Ti (mg L-1)
f)
V (mg L-1)
g)
Cr (mg L-1)
h)
Mn (mg L-1)
i)
Fe (mg L-1)
j)
Co (mg L-1)
k)
Ni (mg L-1)
l)
Cu (mg L-1)
m)
Zn (mg L-1)
n)
As (mg L-1)
o)
Se (mg L-1)
p)
Rb (mg L-1)
q)
Sr (mg L-1)
r)
Mo (mg L-1)
s)
Cd (mg L-1)
t)
Sb (mg L-1)
u)
Cs (mg L-1)
v)
Ba (mg L-1)
w)
Tl (mg L-1)
x)
Pb (mg L-1)
y)
U (mg L-1)
Cl (mg L-1)
Thermal waters (TDS < 4 g L-1)
Thermal waters (TDS > 4 g L-1)
Cold gas-rich waters
Acidic waters
Hyperalkaline waters
Sea water ratio
