# Supplementary material for: Characterization of trace elements in thermal and mineral waters of Greece
Source: Environ Sci Pollut Res Int. 2023 Jun 3;30(32):78376–93. doi: 10.1007/s11356-023-27829-x (PMC10313562; doi:10.1007/s11356-023-27829-x)
Supplement: Supplementary file 3 — Supplementary file3 (PDF 2642 KB) [file 11356_2023_27829_MOESM3_ESM.pdf]

Supplementary material

Figure S3

Geographical distribution of a) Cr, b) Ni, c) Mn, d) Fe, e) As, f) Sb, g) Ba, h) Pb and i) U in the thermal and mineral waters of Greece. Red dashed line represents the South Aegean Active Volcanic Arc (SAAVA). Basemap by *Google Maps. Google, 2015*.

Characterization of trace elements in thermal and mineral waters of Greece

Environmental Science and Pollution Research

Lorenza Li Vigni[1], Kyriaki Daskalopoulou[2,3], Sergio Calabrese[1,4], Konstantinos Kyriakopoulos[5], Sergio Bellomo[4], Lorenzo Brusca[4], Filippo Brugnone[1], Walter D'Alessandro[4*]

1) University of Palermo, DiSTeM, via Archirafi 36, Palermo, Italy
2) University of Potsdam, Institute of Geosciences, Karl-Liebknecht-Str. 24-25, Potsdam-Golm, Germany.
3) GeoForschungs Zentrum, Physics of Earthquakes and Volcanoes, Helmholtzstraße 6/7, Potsdam, Germany
4) Istituto Nazionale di Geofisica e Vulcanologia, sezione di Palermo, via Ugo La Malfa 153, Italy
5) National and Kapodistrian University of Athens, Faculty of Geology and Geoenvironment, Panestimioupolis, Ano Ilissia, Greece

corresponding author: walter.dalessandro@ingv.it

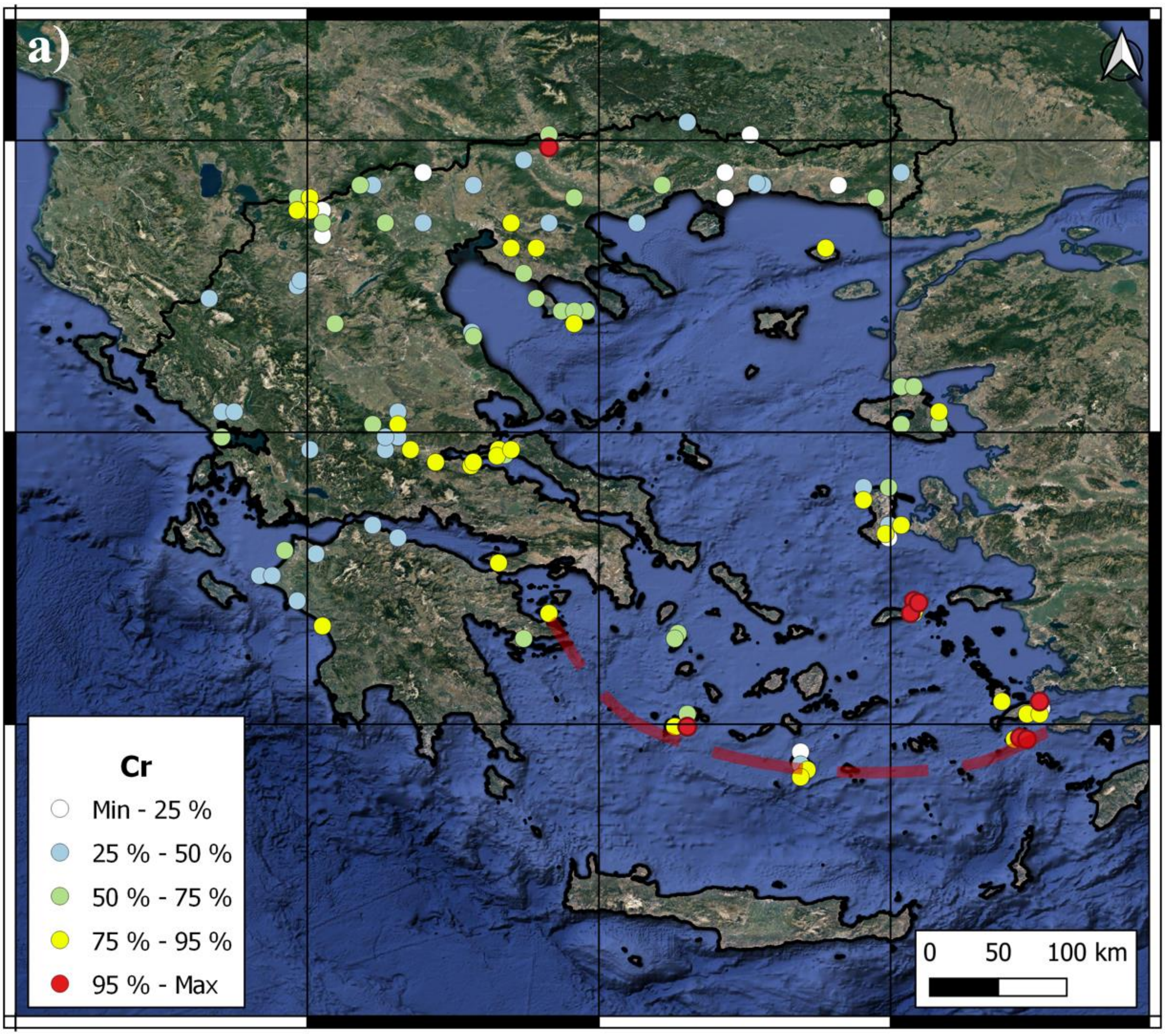
a)
Cr
Min - 25 %
25 % - 50 %
50 % - 75 %
75 % - 95 %
95 % - Max
0 50 100 km

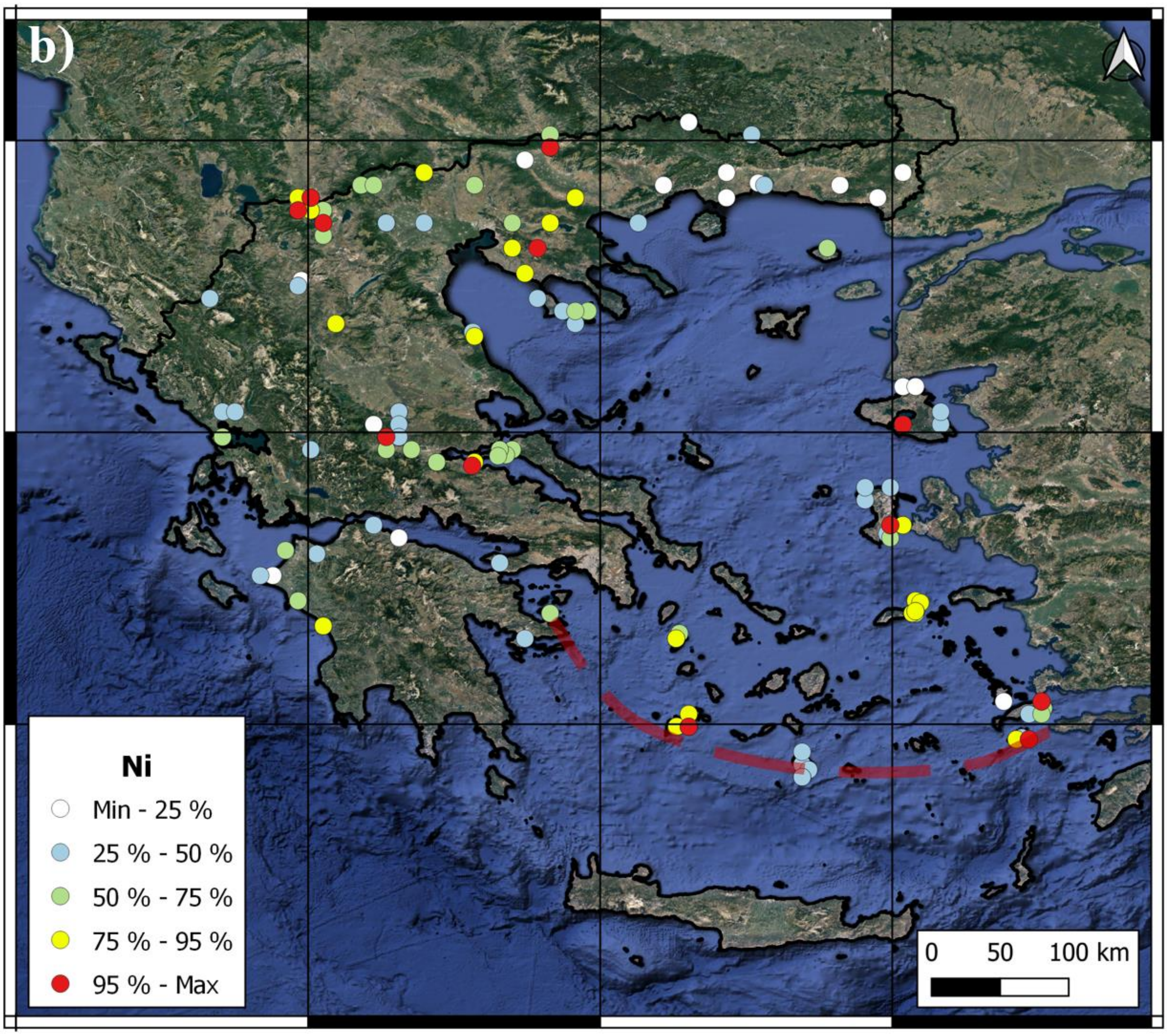
b)
Ni
Min - 25 %
25 % - 50 %
50 % - 75 %
75 % - 95 %
95 % - Max
0 50 100 km

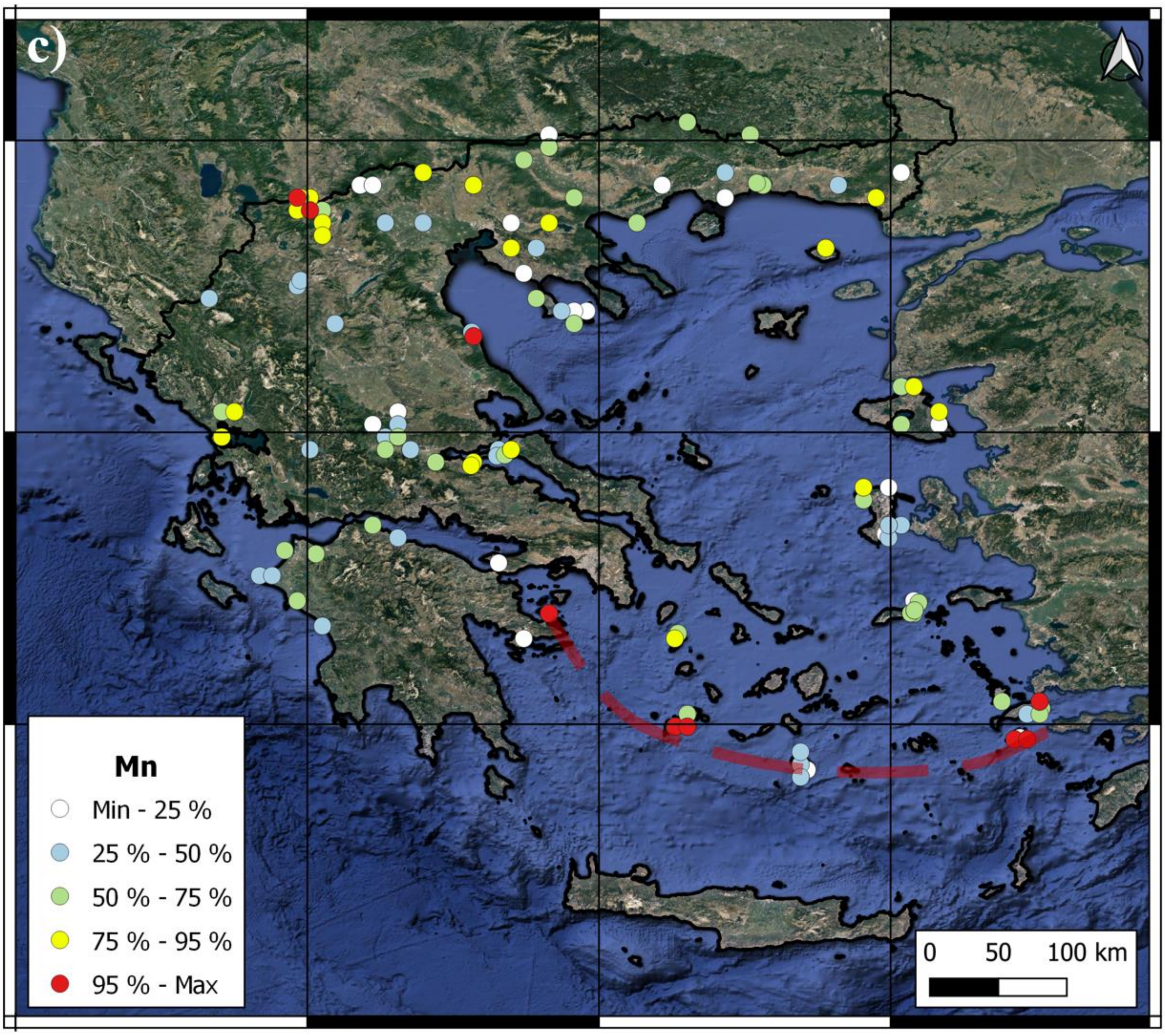
c)
Mn
Min - 25 %
25 % - 50 %
50 % - 75 %
75 % - 95 %
95 % - Max
0 50 100 km

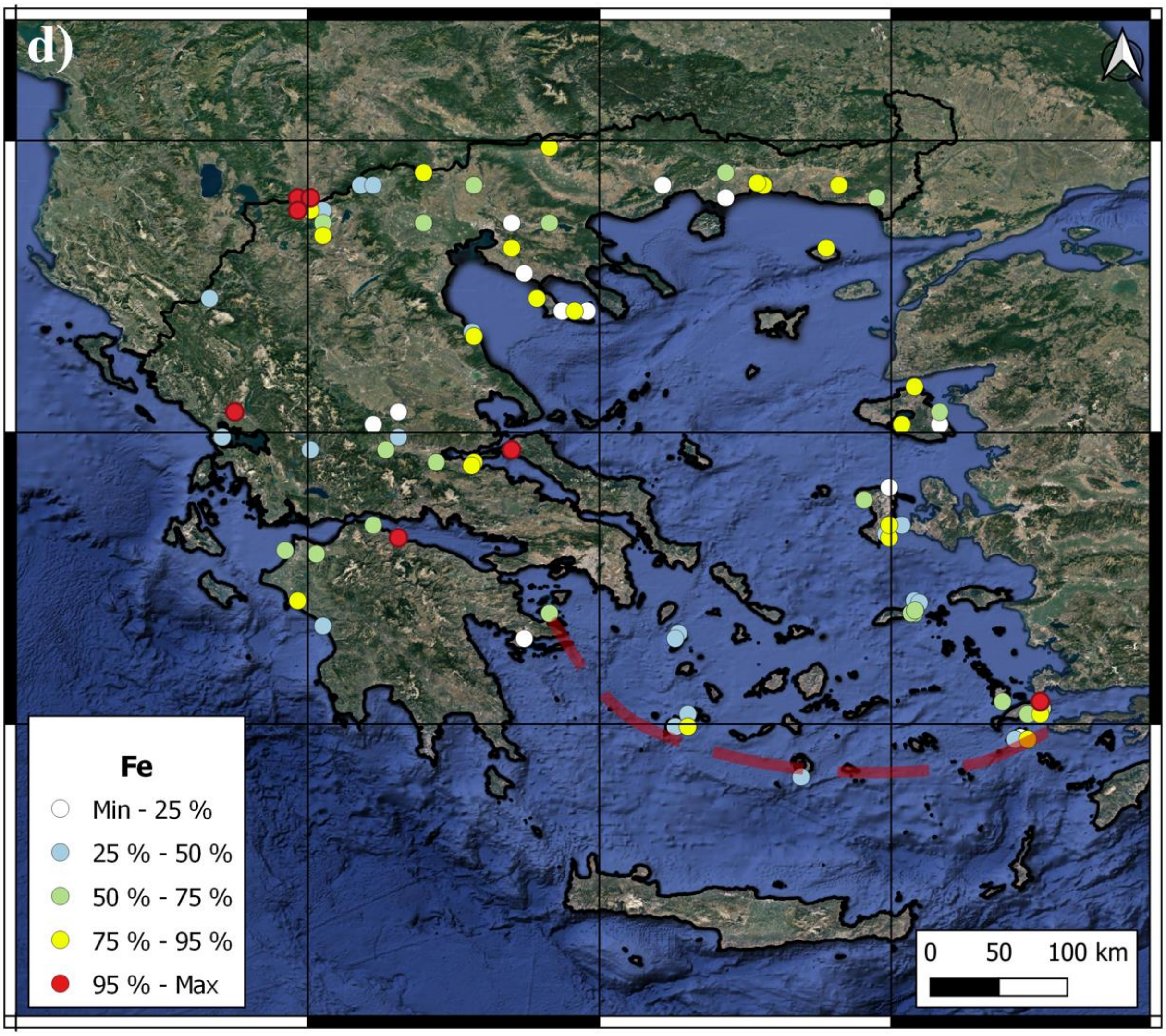
d)
Fe
Min - 25 %
25 % - 50 %
50 % - 75 %
75 % - 95 %
95 % - Max
0 50 100 km

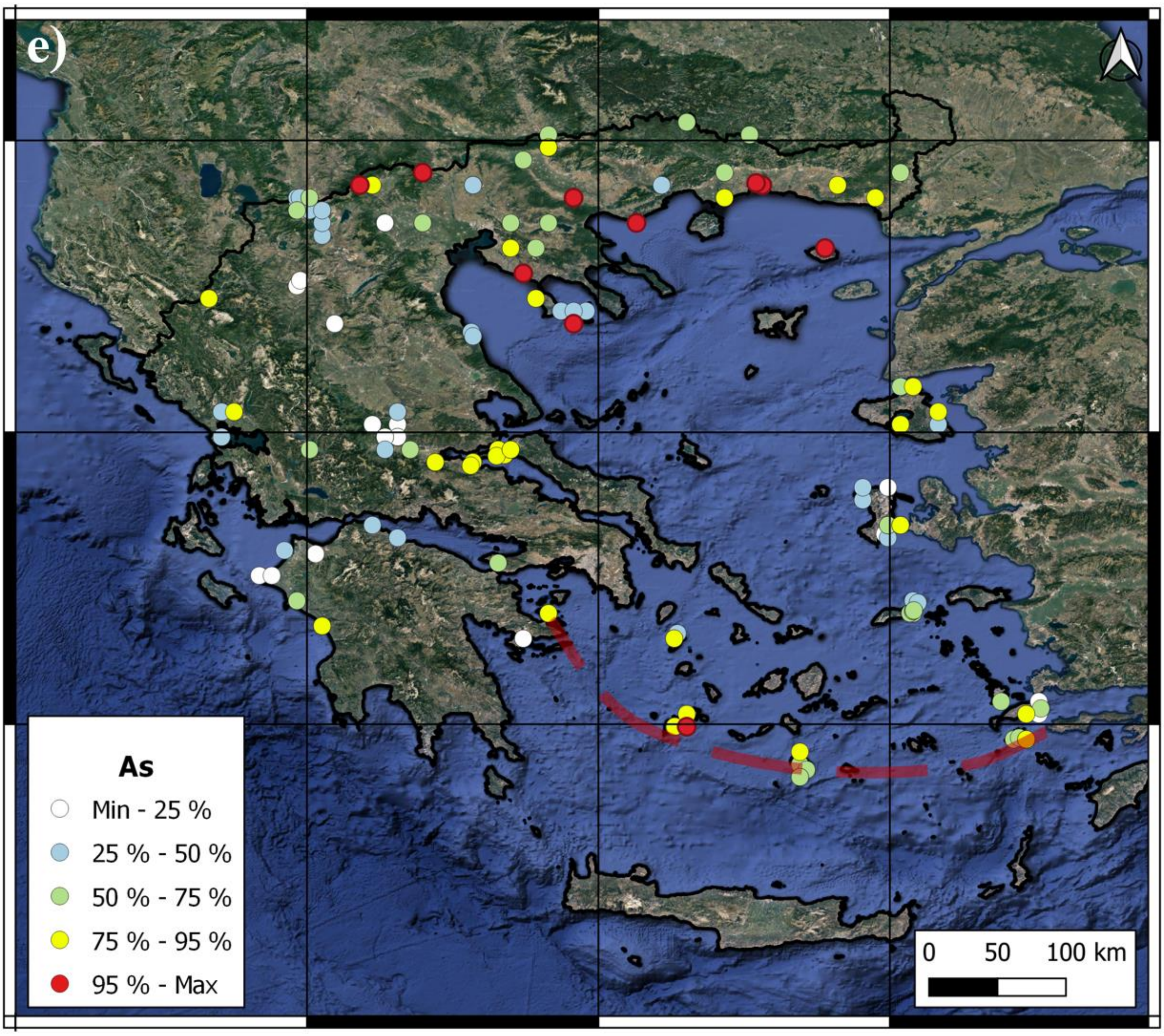
e)
As
Min - 25 %
25 % - 50 %
50 % - 75 %
75 % - 95 %
95 % - Max
0
50
100 km

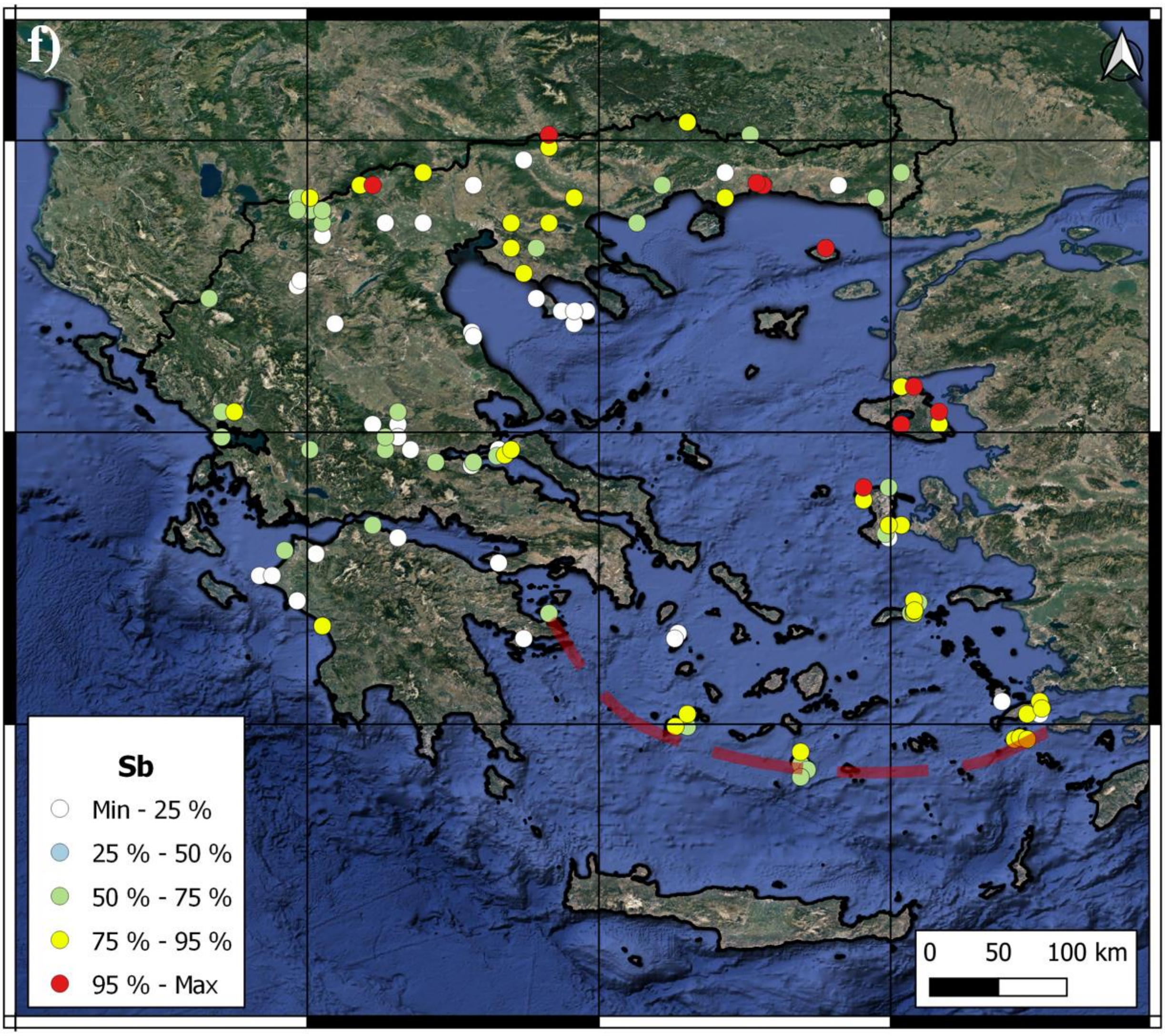
f)
Sb
Min - 25 %
25 % - 50 %
50 % - 75 %
75 % - 95 %
95 % - Max
0 50 100 km

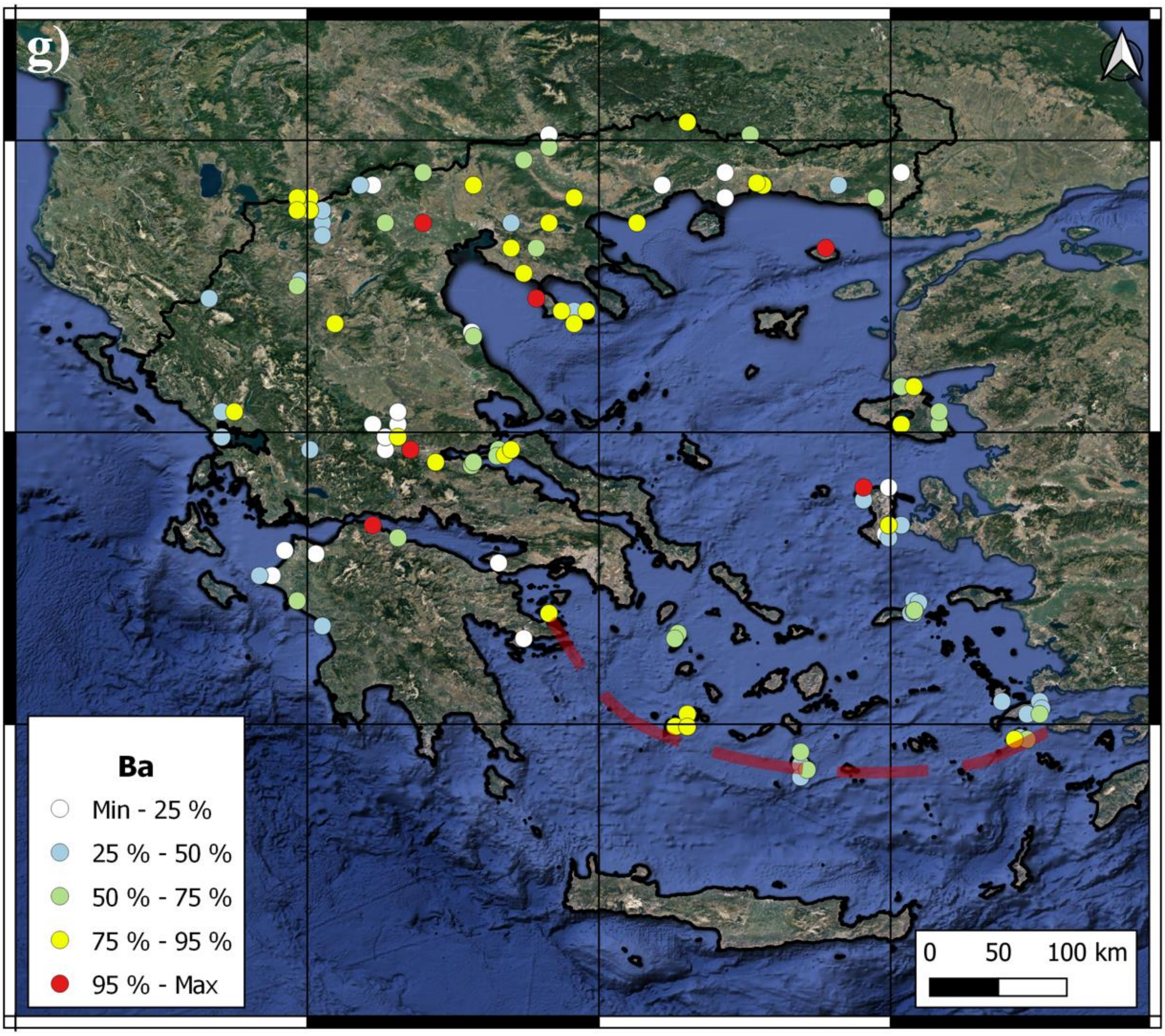
g)
Ba
Min - 25 %
25 % - 50 %
50 % - 75 %
75 % - 95 %
95 % - Max
0 50 100 km

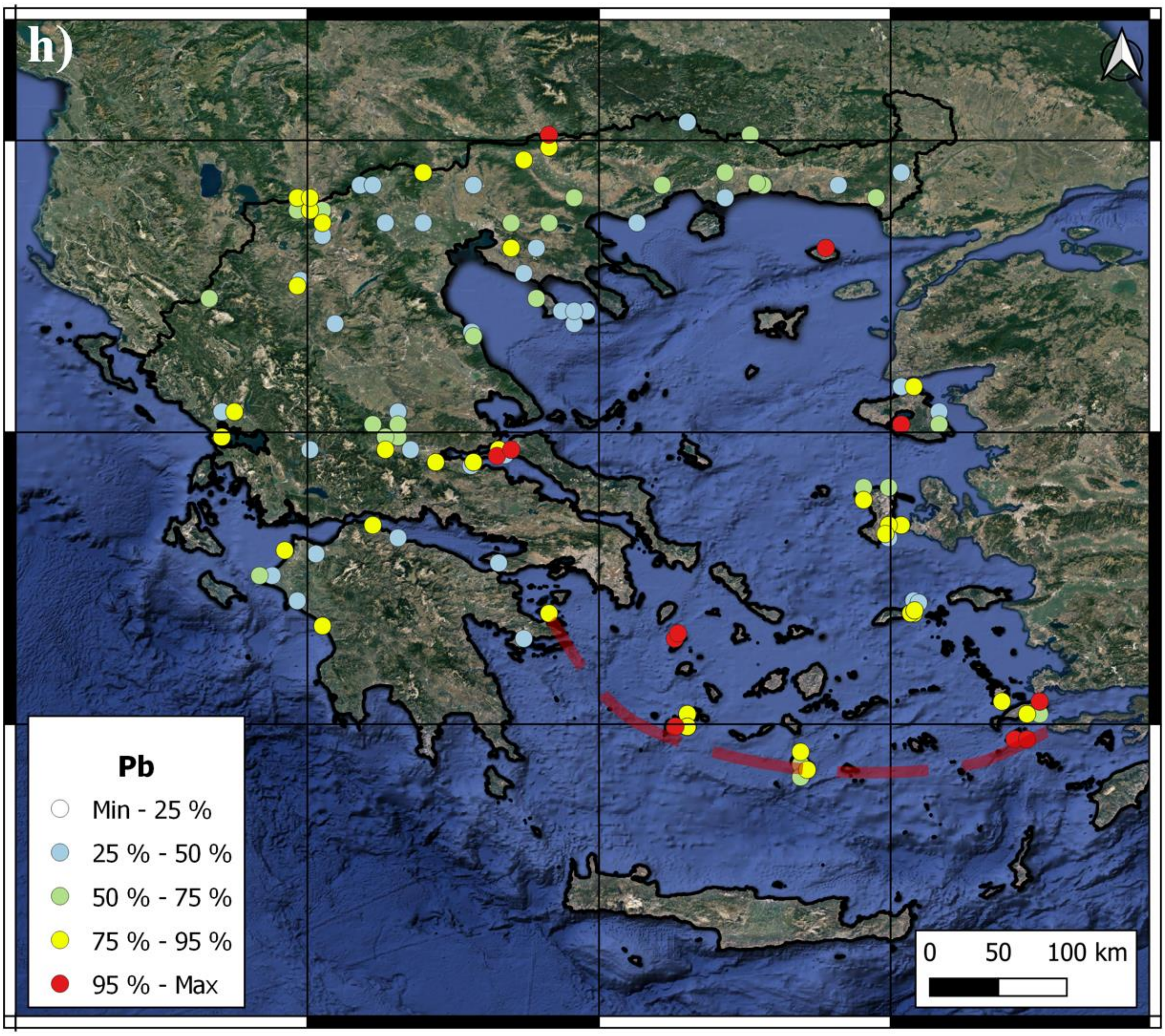

h)
Pb
Min - 25 %
25 % - 50 %
50 % - 75 %
75 % - 95 %
95 % - Max
0
50
100 km

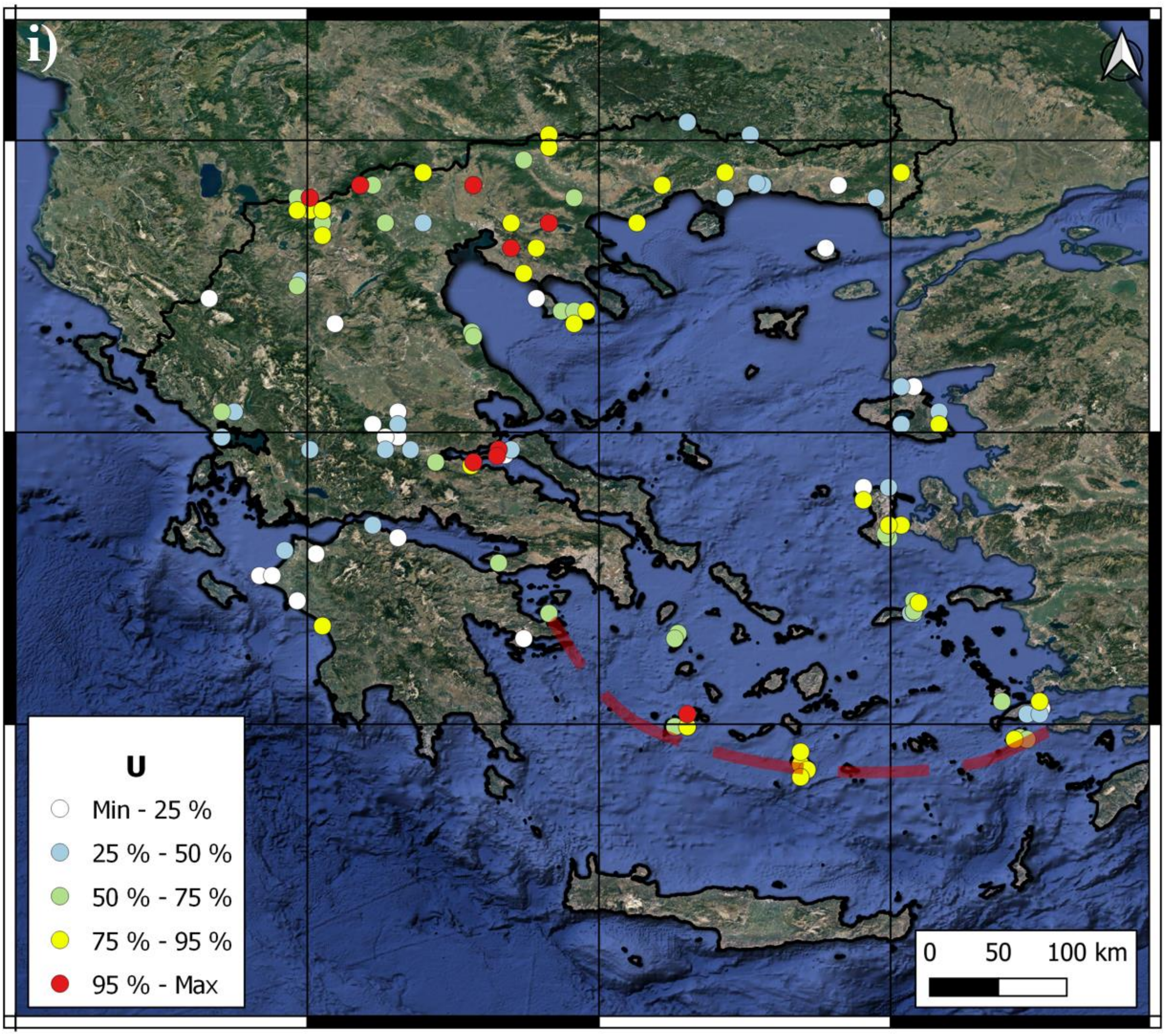

i)
U
Min - 25 %
25 % - 50 %
50 % - 75 %
75 % - 95 %
95 % - Max
0 50 100 km
